# Splicing factor SRSF6 mediates pleural fibrosis

**DOI:** 10.1172/jci.insight.146197

**Published:** 2021-05-24

**Authors:** Li-Mei Liang, Liang Xiong, Pei-Pei Cheng, Shuai-Jun Chen, Xiao Feng, Ya-Ya Zhou, Qian Niu, Meng Wang, Qianlan Chen, Lin-Jie Song, Fan Yu, Xin-Liang He, Fei Xiang, Xiaorong Wang, Hong Ye, Wan-Li Ma

**Affiliations:** 1 Department of Respiratory and Critical Care Medicine, Union Hospital, Tongji Medical College, Huazhong University of Science and Technology, Wuhan, China.; 2 Key Laboratory of Respiratory Diseases, National Health Commission of China, Wuhan, China.; 3 Department of Pathophysiology, School of Basic Medicine, Tongji Medical College, and; 4 Department of Radiology, Tongji Hospital, Tongji Medical College, Huazhong University of Science and Technology, Wuhan, China.

**Keywords:** Pulmonology, Fibrosis, Mouse models

## Abstract

Pleural fibrosis is defined as an excessive deposition of extracellular matrix that results in destruction of the normal pleural tissue architecture and compromised function. Tuberculous pleurisy, asbestos injury, and rheumatoid pleurisy are main causes of pleural fibrosis. Pleural mesothelial cells (PMCs) play a key role in pleural fibrosis. However, detailed mechanisms are poorly understood. Serine/arginine-rich protein SRSF6 belongs to a family of highly conserved RNA-binding splicing-factor proteins. Based on its known functions, SRSF6 should be expected to play a role in fibrotic diseases. However, the role of SRSF6 in pleural fibrosis remains unknown. In this study, SRSF6 protein was found to be increased in cells of tuberculous pleural effusions (TBPE) from patients, and decellularized TBPE, bleomycin, and TGF-β1 were confirmed to increase SRSF6 levels in PMCs. In vitro, SRSF6 mediated PMC proliferation and synthesis of the main fibrotic protein COL1A2. In vivo, SRSF6 inhibition prevented mouse experimental pleural fibrosis. Finally, activated SMAD2/3, increased SOX4, and depressed miRNA-506-3p were associated with SRSF6 upregulation in PMCs. These observations support a model in which SRSF6 induces pleural fibrosis through a cluster pathway, including SRSF6/WNT5A and SRSF6/SMAD1/5/9 signaling. In conclusion, we propose inhibition of the splicing factor SRSF6 as a strategy for treatment of pleural fibrosis.

## Graphical abstract

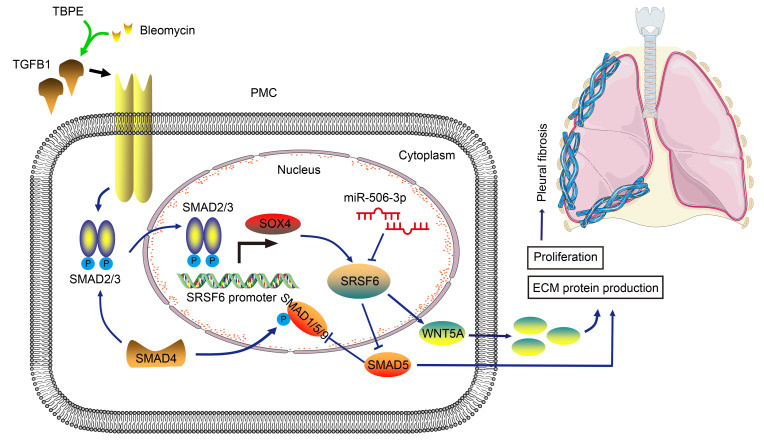

## Introduction

Pleural fibrosis is defined as an excessive deposition of extracellular matrix (ECM) that results in destruction of the normal pleural tissue architecture and compromised function. Tuberculous pleurisy, asbestos injury, and rheumatoid pleurisy are major causes of pleural fibrosis (1). In cases of tuberculous pleurisy, when tuberculous pleural effusions (TBPE) resolve, pleural thickening occurs in about one-half of patients (2). In some severe cases, the progression of pleural fibrosis leads to lung entrapment, resulting in dyspnea and respiratory failure. Although corticosteroids could be useful in fibrotic diseases, there is insufficient evidence to support their use to prevent or diminish the development of pleural fibrosis in TBPE (3). Thus, there is currently no effective medication for pleural fibrosis. Therefore, understanding the detailed mechanisms of pleural fibrosis is an important unmet need, which could lead to the identification of new targets for treatment of this condition.

The pleura are composed of a monolayer of pleural mesothelial cells (PMCs) and a thin basement membrane supported by connective tissue, blood vessels, and lymphatics. PMCs secrete glycosaminoglycans and other surfactant-like molecules to lubricate the pleural surface, providing a smooth lubricating surface for movement of the lung during inspiration and expiration. In addition, PMCs can release proinflammatory and antiinflammatory mediators and synthesize growth factors and ECM proteins to assist pleural membrane repair (4). However, dysfunctional pleural membrane repair is associated with pleural fibrosis (5). Functional or dysfunctional pleural repair mainly depends on the proteins that are synthesized and released from PMCs. TGF-β, basic fibroblast growth factor (bFGF), and PDGF are considered to play roles in the pathogenesis of pleural fibrosis (5); however, their mechanistic involvement is thought to be more complicated than simple changes in TGF-β, bFGF, or PDGF. Thus, a more detailed understanding of the molecular pathogenesis of pleural fibrosis is required.

Alternative splicing is an important mechanism to regulate gene expression (6). Moreover, this regulatory mechanism, leading to the expression of multiple isoforms of a single gene, is a major contributor to proteome complexity. The serine/arginine-rich (SR) protein SRSF6 (SRp55) belongs to a family of highly conserved RNA-binding splicing-factor proteins (7), with 1 or 2 RNA-recognition motifs and a C-terminal SR domain (8). Previously, it was reported that SRSF6 participated in fibrosis in patients with systemic sclerosis (9). However, the role of SRSF6 in pleural fibrosis is unknown. In this study, using clinical samples, cell models, and animal models, we investigated the role of SRSF6 in pleural fibrosis as well as underlying mechanisms.

## Results

### Tuberculous pleural effusion increased SRSF6 and COL1A2 protein in PMCs.

**Figure 1:**
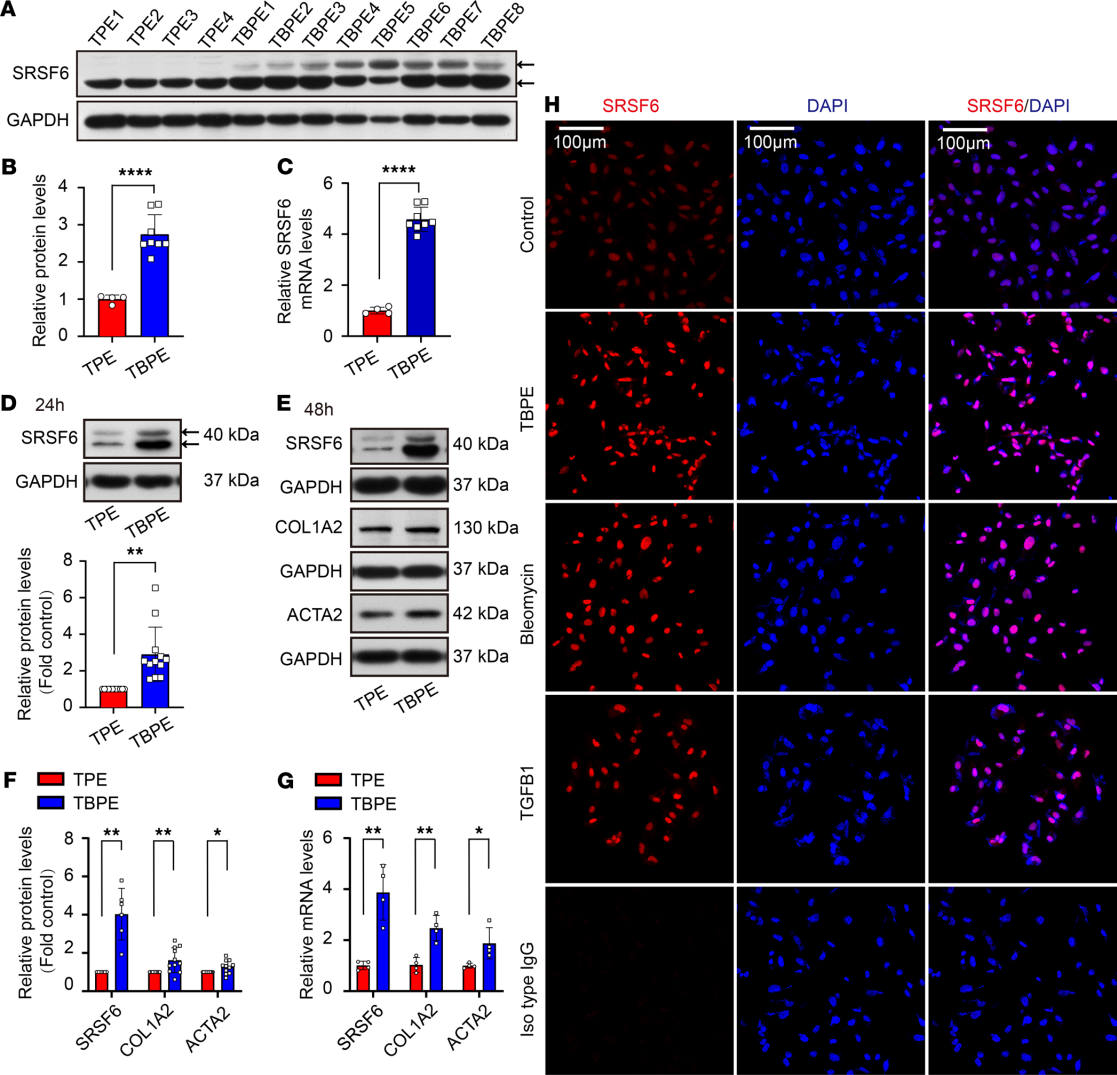
Tuberculous pleural effusion increased SRSF6 and COL1A2 protein in pleural mesothelial cells. (**A**–**C**) Cells were collected from tuberculous pleural effusion (TBPE) (*n =* 8) or transudative pleural effusion (*n =* 4). Relative SRSF6 protein levels and mRNA were investigated by Western blotting (**A** and **B**) and quantitative real-time PCR (qRT-PCR) (**C**). (**D**–**G**) Human pleural mesothelial cells (PMCs) were incubated with TBPE (5%), TPE (5%) for 24 or 48 hours, after which intracellular protein levels of SRSF6, COL1A2, and ACTA2 were measured by Western blotting. (**D**) *n* = 12; (**F**) *n* = 6 (SRSF6), *n* = 11 (COL1A2), *n* = 12 (ACTA2). (**G**) mRNA levels of SRSF6, COL1A2, and ACTA2 were detected by RT-qPCR at 48 hours; *n* = 4. (**H**) Human PMCs were incubated with TBPE (5%), bleomycin (0.2 μg/mL), or TGF-β1 (5 ng/mL). After 24 hours, SRSF6 protein was detected by immunofluorescence staining and nuclei with DAPI staining. Scale bar: 100 μm. Original magnification, ×200. Data are shown as mean ± SEM of *n* individual experiments. *P* values were determined by unpaired Student’s *t* test. *P < 0.05, **P < 0.01, ****P < 0.0001 (versus TPE).

SRSF6 protein and mRNA levels were explored in clinical samples and found to be significantly higher in cells from patients TBPE than that from transudative pleural effusion (TPE) (Figure 1, A–C). We next used decellularized TBPE to treat cultured PMCs and showed changes in SRSF6 protein and mRNA by Western blotting, quantitative real-time PCR (qRT-PCR), and immunofluorescence staining. As shown in Figure 1, D–H, SRSF6 was increased in PMCs treated with TBPE for 24 or 48 hours. Moreover, fibrosis-related proteins, COL1A2 (collagen type 1 α 2), and ACTA2 (α actin 2) synthesis was also upregulated by TBPE (Figure 1, E–G). Furthermore, immunohistochemical and immunofluorescence staining of clinical pleural biopsy tissues revealed that SRSF6 was expressed in tuberculous pleurisy tissues (Supplemental Figures 1 and 2; supplemental material available online with this article; https://doi.org/10.1172/jci.insight.146197DS1). These results indicated that SRSF6 protein is increased in tuberculous pleuritis and that TBPE can induce SRSF6 and COL1A2 expression in PMCs.

### Bleomycin and TGF-β1 increased SRSF6 and COL1A2 protein in PMCs.

Bleomycin is a known inducer of pulmonary and pleural fibrosis, whereas TGF-β1 is a classical growth factor implicated in fibrotic diseases. To further investigate the role of SRSF6 in pleural fibrosis, bleomycin and TGF-β1 were used to stimulate cultured PMCs. As shown in Figure 1H and Supplemental Figure 3, bleomycin induced increased in protein and mRNA levels of SRSF6, COL1A2, and ACTA2. Moreover, expression of SRSF6 and COL1A2 protein induced by TGF-β1 was dose dependent (Supplemental Figure 3, D–F).

### SRSF6 mediated TBPE-, bleomycin-, and TGF-β1–induced COL1A2 synthesis in PMCs.

**Figure 2:**
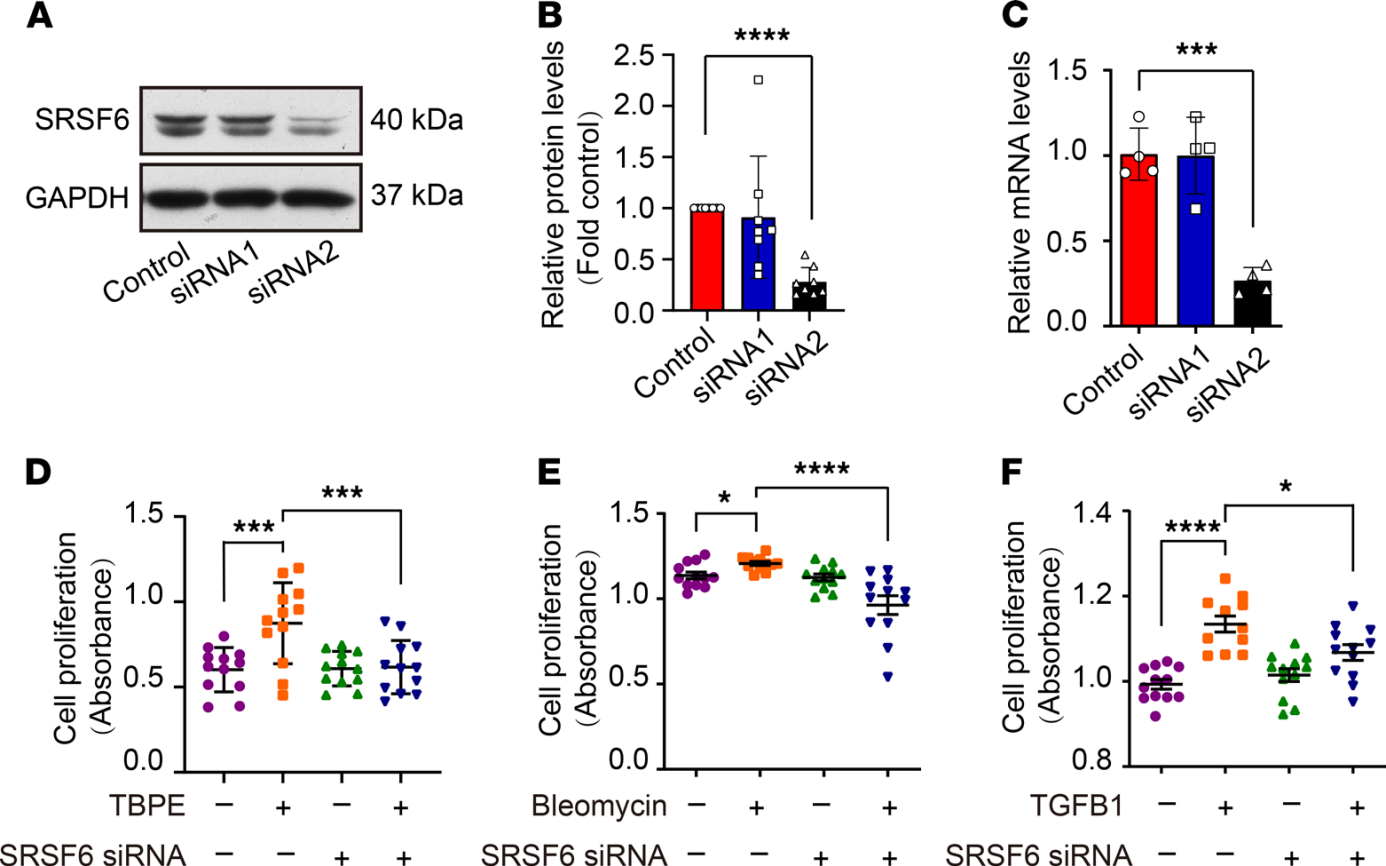
SRSF6 knock down by siRNA prevented TBPE-, bleomycin-, and TGF-β1–induced PMC proliferation in vitro. (**A**–**C**) Human PMCs were transfected with control or siRNAs specific for SRSF6 (siRNA1 and siRNA2) for 48 hours. SRSF6 protein and siRNA knockdown levels were assessed by Western blotting (**A** and **B**) and qRT-PCR (**C**). Data are shown as mean ± SEM of *n* individual experiments. (**B**) *n* = 8, (**C**) *n* = 4. ***P < 0.001, ****P < 0.0001 (versus control group) (Student’s *t* test). (**D**–**F**) After transfection with SRSF6 siRNA2 or negative control siRNA, PMCs were incubated with or without TBPE (5%), bleomycin (0.2 μg/mL), or TGF-β1 (5 ng/mL) for 24 hours, and cell proliferation was measured by CCK-8 assay. Data are shown as mean ± SEM of *n* individual experiments. (**D**–**F**) *n* = 12. *P < 0.05, ***P < 0.001, ****P < 0.0001 (1-way ANOVA followed by the Bonferroni’s test).

**Figure 3:**
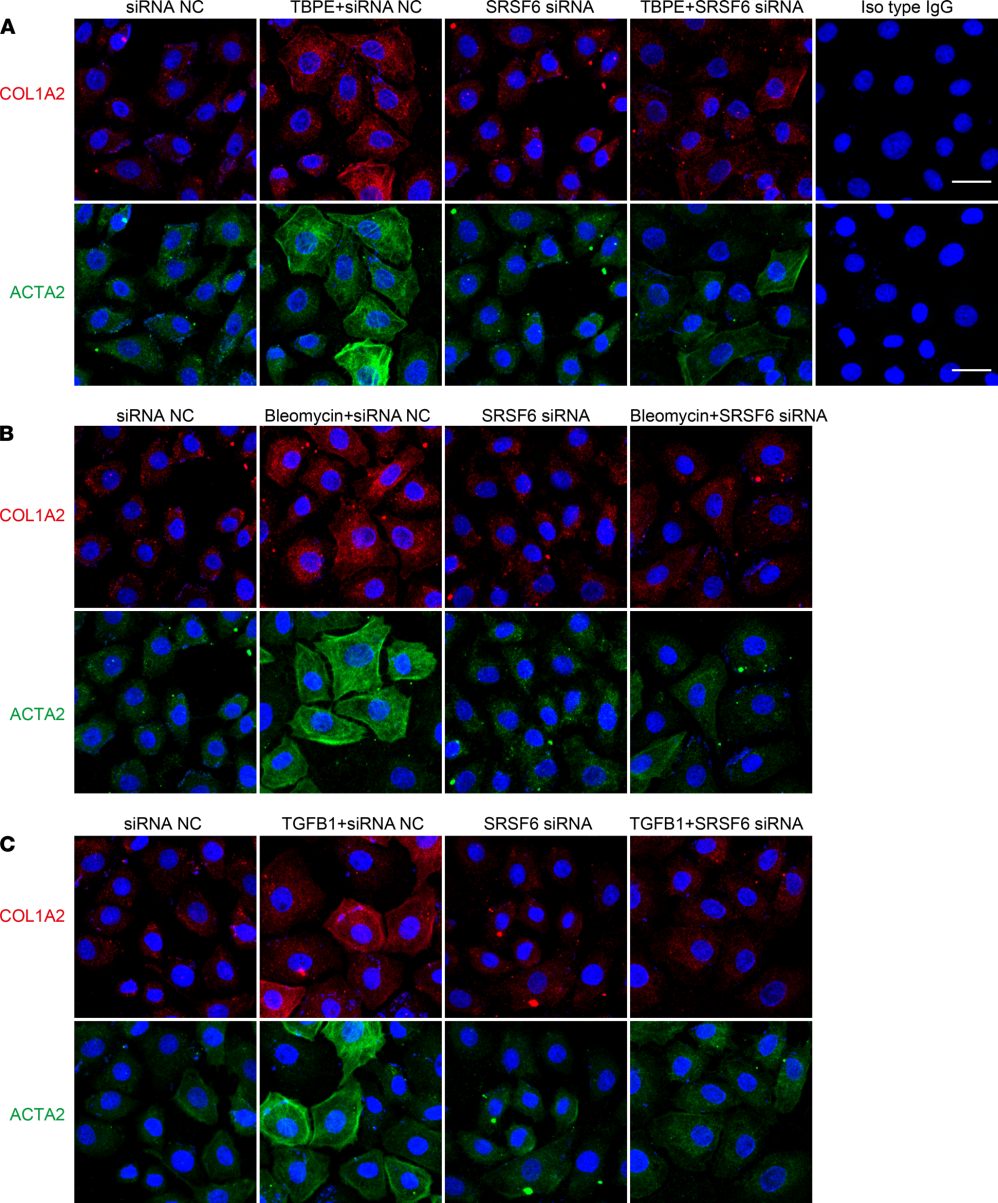
SRSF6 knock down by siRNA suppressed TBPE-, bleomycin- and TGF-β1–induced COL1A2 and ACTA2 synthesis in PMCs. After transfection with SRSF6 siRNA or negative control siRNA (siRNA NC), PMCs were incubated with or without (**A**) TBPE (5%), (**B**) bleomycin (0.2 μg/mL), or (**C**) TGF-β1 (5 ng/mL) for 48 hours, after which COL1A2 and ACTA2 protein was detected by immunofluorescence staining. Red, COL1A2; green, ACTA2; and blue, DAPI. Scale bar: 50 μm. Original magnification, ×630.

To understand whether COL1A2 synthesis is regulated by SRSF6 in PMCs, SRSF6 siRNA was used to knock down its protein level. As shown in Figure 2, A–C, 2 candidates of human SRSF6 siRNAs were tested in human PMCs, and siRNA2 was shown to depress SRSF6 protein expression by approximately 70%. This SRSF6 siRNA was used in subsequent experiments. As shown in Figure 2, D–F, and Supplemental Figure 4, SRSF6 siRNA prevented PMC proliferation induced by TBPE, bleomycin, or TGF-β1. TBPE increased protein and mRNA levels of COL1A2 and ACTA2 in PMCs, but this was prevented by SRSF6 siRNA (Figure 3A and Supplemental Figure 5, A–C). Similar results were also found in rat primary PMCs (Supplemental Figure 6). TBPE, bleomycin, and TGF-β1 induced upregulation of COL1A2 and ACTA2 mRNA as well as their proteins, SRSF6 siRNA attenuated mRNA and protein levels increased by bleomycin or TGF-β1 (Figure 3 and Supplemental Figure 5, A–I). These data indicated that SRSF6 mediated TBPE-, bleomycin-, and TGF-β1–induced cell proliferation, COL1A2 and ACTA2 synthesis in PMCs.

### SRSF6 inhibition prevented pleural fibrosis in a mouse model.

**Figure 4:**
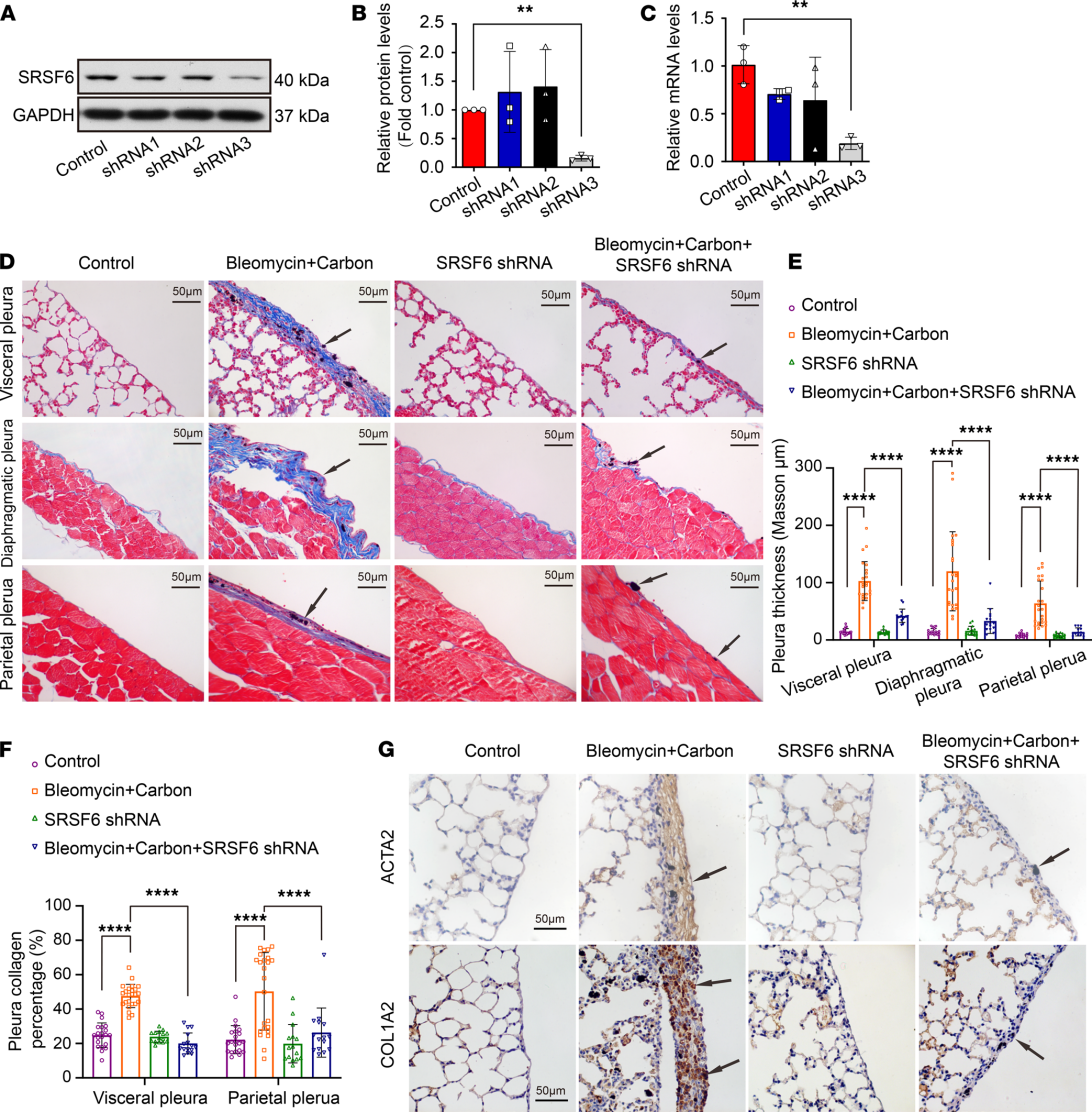
SRSF6 knock down attenuated bleomycin-induced pleural fibrosis in vivo. (**A**–**C**) Mouse alveolar epithelial cells, MLE-12, were infected with lentivirus expressing control or specific shRNA against SRSF6 (shRNA1, shRNA2, and shRNA3) for 48 hours. SRSF6 protein and mRNA levels were assessed by Western blotting (**A** and **B**) or qRT-PCR (**C**). Data are shown as mean ± SEM of *n* individual experiments, *n =* 3 (**B** and **C**), **P < 0.01 (versus control group) (Student’s *t* test). (**D**–**G**) The mouse pleural fibrosis model was induced by intrapleural injections of bleomycin plus carbon particles. Lentivirus expressing shRNA directed against SRSF6 or scrambled sequence shRNA were administrated by intrapleural injection at a dose of 2 × 10^6^ TU on days 4, 7, and 10. All mice were euthanized at day 21, and then tissues were taken for analysis. (**D**) Representative Masson’s trichrome staining images of visceral pleura from lung sections, parietal pleura from chest wall, and diaphragm sections. Original magnification, ×400. (**E**) Changes in pleural thickness. (**F**) Changes in collagen percentages of visceral and parietal pleura. (**G**) Representative IHC staining for COL1A2 and ACTA2 on visceral pleura from lung sections. Scale bar: 50 μm. Original magnification, ×400. Data are shown as mean ± SEM of *n* individual experiments. *n =* 7 (control shRNA group), *n =* 8 (control shRNA and bleomycin plus carbon particles group), *n =* 6 (SRSF6 shRNA3 group, SRSF6 shRNA3 and bleomycin plus carbon particles group). ****P < 0.0001 (1-way ANOVA followed by the Bonferroni’s test).

Pleural fibrosis in mice was induced by intrapleural injections of bleomycin plus carbon particles, as described in Methods. Lentivirus mouse-specific SRSF6 shRNAs were tested using a mouse alveolar epithelial cell, MLE-12, and shRNA3 was shown to knock down protein and mRNA by approximately 80% (Figure 4, A–C). This shRNA was selected for administration by intrapleural injection in the mouse model. As shown in Figure 4, D–F, and Supplemental Figures 7 and 8, bleomycin plus carbon induced mouse pleural thickening and fibrosis in the visceral, parietal, and diaphragmatic pleura. Similar to changes in cultured PMCs, SRSF6 protein and mRNA were highly expressed in the pleural fibrosis (Supplemental Figure 7), and COL1A2 and ACTA2 protein dramatically increased in the pleural fibrosis models (Figure 4, D–G and Supplemental Figure 8). More importantly, pleural fibrosis in mice was obviously attenuated by intrapleural administration of lentivirus encoding SRSF6 shRNA (Figure 4, D–G and Supplemental Figures 7 and 8). These results revealed that SRSF6 knock down attenuated pleural fibrosis in the experimental mouse model.

### SMAD2/3/SOX4 regulated SRSF6 expression in PMCs.

**Figure 5:**
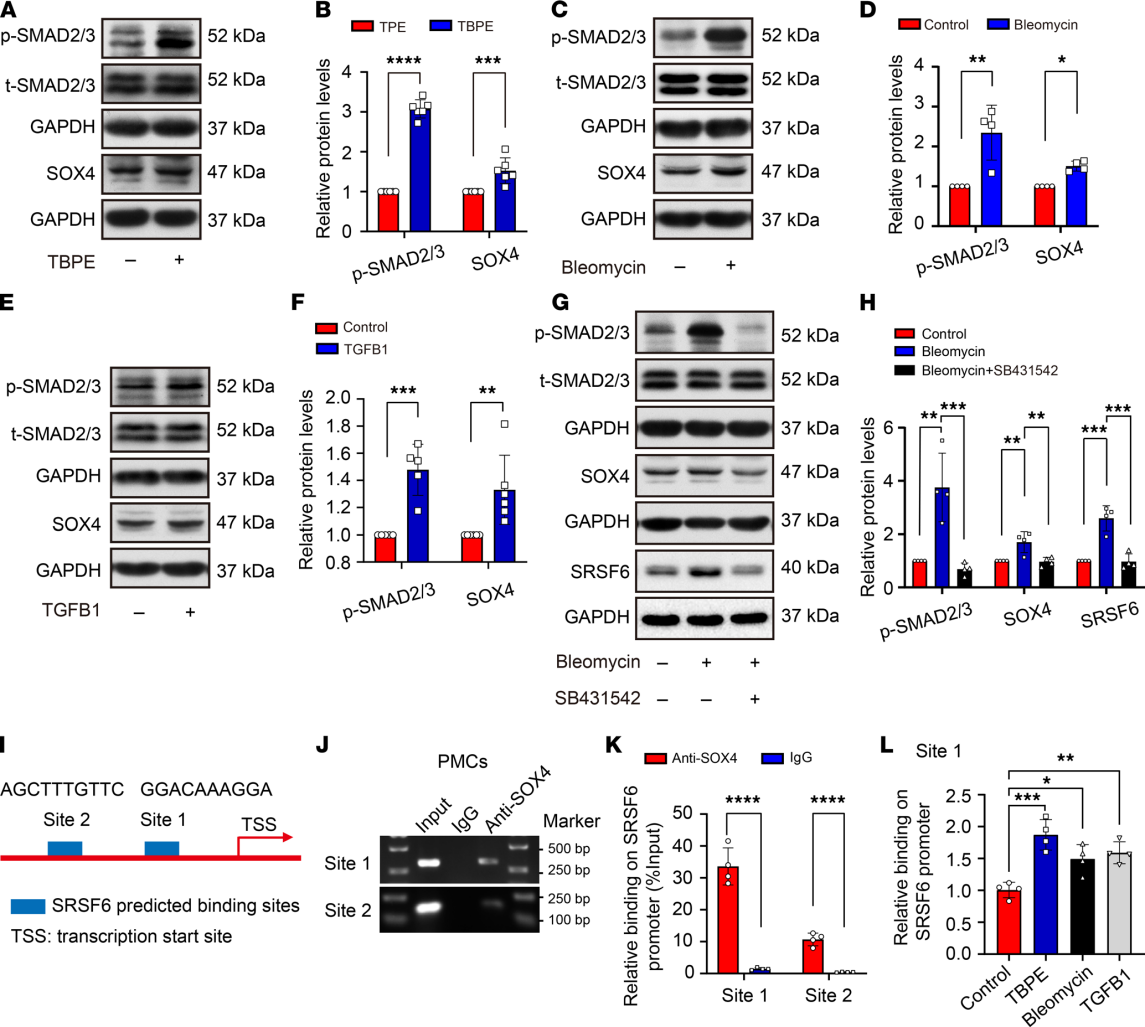
SMAD2/3 and SOX4 upregulated SRSF6 expression in PMCs. (**A**–**F**) Human PMCs were incubated with or without TBPE (5%), bleomycin (0.2 μg/mL), and TGF-β1 (5 ng/mL) for 24 hours, after which intracellular protein levels of p-SMAD2/3 (phosphorylated), t-SMAD2/3 (total), and SOX4 were measured by Western blotting (**A**, **C**, and **E**). Bar graphs revealed changes in the relative ratio of p-SMAD2/3 to t-SMAD2/3 and GAPDH, or SOX4 to GAPDH (**B**, **D**, and **F**). (**B)** *n =* 6; (**D**) *n =* 4; and (**F**) *n =* 5 (p-SMAD2/3), *n =* 6 (SOX4). (**G** and **H**) PMCs were treated with bleomycin (0.2 μg/mL) in the presence or absence of SB413542 (10 ng/mL) for 24 hours, after which intracellular protein levels were measured by Western blotting. *n =* 4. (**I**) A schematic diagram illustrating the 2 putative SOX4 binding sites in the *SRSF6* promoter. (**J** and **K**) ChIP assay of PMCs. Binding of SOX4 to the 2 sites was confirmed by PCR (**J**) and qPCR (**K**) with primers specific for the 2 sites, *n* = 4. (**L**) ChIP assay of PMCs treated with or without TBPE (5%), bleomycin (0.2 μg/mL), and TGF-β1 (5 ng/mL) for 24 hours. SOX4 binding to site 1 was confirmed by qPCR with specific primers, *n* = 4. Data are shown as mean ± SEM of *n* individual experiments. *P < 0.05, **P < 0.01, ***P < 0.001, ****P < 0.0001. *P* values were determined by Student’s *t* test (**B**, **D**, **F**, and **K**) or 1-way ANOVA (**H** and **L**).

Next, upstream signaling, which regulate SRSF6 expression in PMCs, was explored. TBPE, bleomycin, and TGF-β1 were each confirmed to activate SMAD2/3 signaling and increase SOX4 protein and mRNA in PMCs (Figure 5, A–F). Furthermore, the TGF-β receptor inhibitor, SB431542, inhibited SMAD2/3 phosphorylation as well as SOX4 expression (Figure 5, G and H). Two putative SOX4 bindings are present in the 5′ proximal region of the *SRSF6* transcriptional start site, and SOX4 binding to these sites was detected by ChIP analysis in PMCs (Figure 5, I–K).Notably, TBPE, bleomycin, and TGF-β1 each induced increased SOX4 binding to site 1 (Figure 5L). Moreover, siRNA-mediated SOX4 knock down inhibited SRSF6 mRNA and protein expression in PMCs (Supplemental Figure 9). In vivo, TGF-β1, SOX4, and SRSF6 were increased in the mouse pleural fibrosis model (Supplemental Figure 10). These data collectively support a model where SMAD2/3 and SOX4 regulate SRSF6 in the context of pleural fibrosis.

### miR-506-3p inhibited SRSF6 expression after transcription.

**Figure 6:**
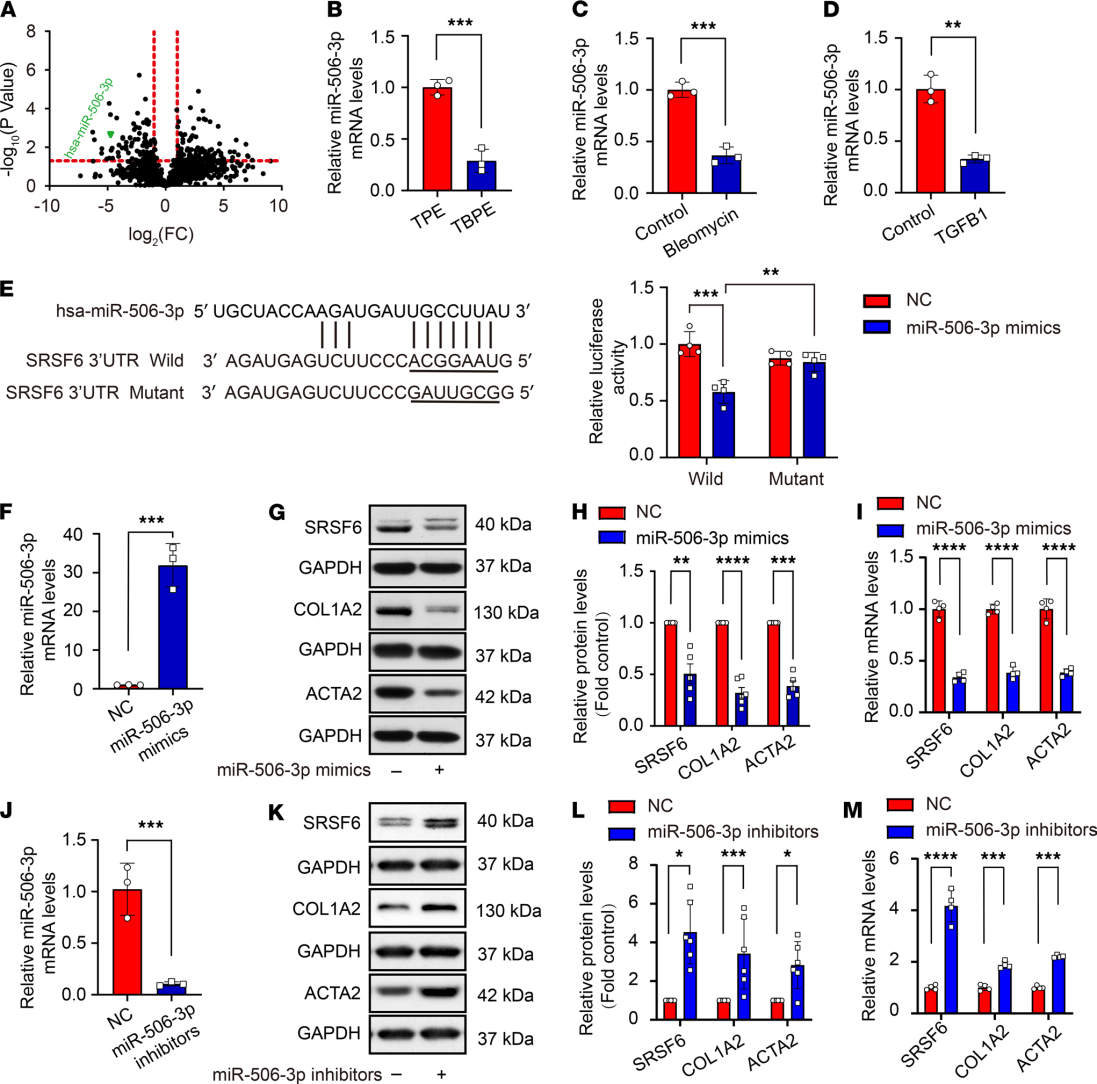
miR-506-3p–regulated SRSF6, COL1A2, and ACTA2 synthesis in human PMCs. (**A**) miRNA profiles were investigated in exosomes from TBPE and TPE. Differentially expressed miRNAs between TBPE and TPE were shown in the volcano plot. hsa-miR-506-3p (marked in green) was lower in TBPE relative to TPE. (**B**–**D**) Human PMCs were treated with TBPE vs. TPE (5%), bleomycin (0.2 μg/mL), or TGF-β1 (5 ng/mL) for 24 hours, after which miR-506-3p expression levels in cells were determined by qRT-PCR and normalized by the U6. Data are mean ± SEM of *n* individual experiments, *n* = 3 (**B**–**D**). **P < 0.01, ***P < 0.001 (unpaired Student’s *t* test). (**E**) Predicted miR-506-3p target sequences in the 3′ UTR of the *SRSF6* mRNA. Sequences of hsa-miR-506-3p and the putative target sequence in the *SRSF6* mRNA (WT) or an engineered mutant of this sequence (mutant) and its effect on the luciferase reporter. Data are shown as mean ± SEM of *n* individual experiments. *n* = 4, **P < 0.01, ***P < 0.001 (2-way ANOVA followed by Tukey’s test). (**F**–**M**) Human PMCs were transfected with miR-506-3p mimics or miR-506-3p inhibitors for 48 hours. miR-506-3p negative control (NC) mimics or NC inhibitors were used as controls. mRNA expression level of miR-506-3p was determined by RT-qPCR (**F** and **J**). The protein expression of SRSF6, COL1A2, and ACTA2 was detected by Western blotting (**G** and **K**). Bar graph depicted changes in relative density of proteins (**H** and **L**). mRNA levels of *SRSF6*, *COL1A2*, and *ACTA2* were detected by RT-qPCR (**I** and **M**). Data are shown as mean ± SEM of *n* individual experiments. (**F)** *n =* 3; (**H)** *n =* 5 (SRSF6), *n =* 6 (COL1A2), and *n =* 5 (ACTA2); (**I**) *n =* 4; (**J**) *n =* 3; (**L**) *n =* 6; and (**M)** *n =* 4. *P < 0.05, **P < 0.01, ***P < 0.001, ****P < 0.0001 (Student’s *t* test).

miRNAs play an important role in the posttranscriptional control of gene expression. We endeavored to identify which miRNAs target *SRSF6* by using TargetScan prediction software. The results indicated that *SRSF6* is highly likely to be the target gene of miR-506-3p. Since cellular fractions from TBPE contained activities capable of promoting fibrosis-like behavior in PMCs, we decided to compare the exosomal miRNA composition of TBPE and TPE. As shown in Figure 6A, hsa-miR-506-3p was reduced in exosomes from TBPE relative to those from TPE. We next determined miR-506-3p levels in PMCs treated by TBPE, bleomycin, or TGF-β1. As shown in Figure 6, B–D, miR-506-3p levels were suppressed in PMCs by TBPE, bleomycin, and TGF-β1.

To determine whether miR-506-3p directly regulates *SRSF6* gene expression, the binding ability of miR-506-3p to the 3′ UTR of *SRSF6* gene was evaluated. miR-506-3p overexpression significantly decreased the luciferase activity of a reporter vector containing the WT *SRSF6* 3′ UTR, but mutation of the presumptive miR-506-3p binding site of this reporter vector abolished the miR-506-3p–associated decrease in the luciferase activity (Figure 6E). Next, the regulation effect of miR-506-3p on SRSF6 expression was tested. miR-506-3p overexpression mimics in PMCs inhibited SRSF6 protein and mRNA expression, as well as COL1A2 and ACTA2 protein in PMCs (Figure 6, F–I). In contrast, antagonizing miR-506-3p with siRNA promoted SRSF6 protein and mRNA expression, and increased COL1A2 and ACTA2 protein and mRNA levels in PMCs (Figure 6, J–M).

Moreover, TPBE, bleomycin, and TGF-β1 increased mRNA and protein levels of SRSF6 in PMCs, but these changes were prevented by miR-506-3p overexpression (Supplemental Figure 11). miR-506-3p also attenuated TBPE-, bleomycin-, and TGF-β1–induced cell proliferation (Supplemental Figure 12) and COL1A2 and ACTA2 synthesis in PMCs (Supplemental Figure 11). These data indicated that depressed miR-506-3p induced increases of SRSF6 expression in PMCs, which was involved in the fibrotic process.

### Downstream roles of SRSF6 in PMCs.

**Figure 7:**
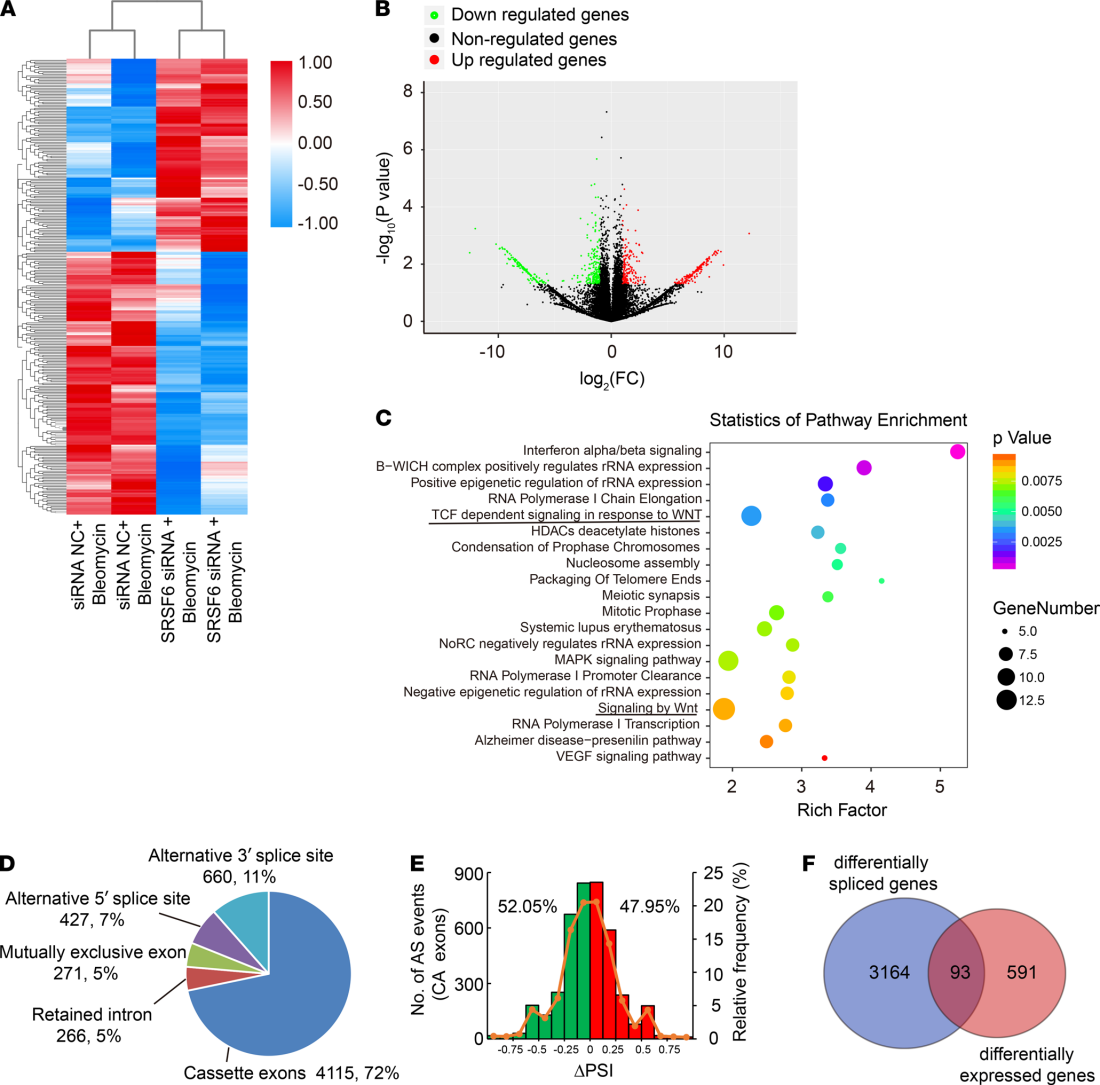
RNA sequencing of bleomycin challenged human PMCs after SRSF6 depletion. (**A**–**C**) After transfection with human SRSF6 siRNA2 or negative control siRNA, PMCs were incubated with bleomycin (0.2 μg/mL) for 24 hours, after which RNA sequencing was performed. (**A**) The heat map of differentially expressed genes, RPKM values were represented by gradient colors and shown for each sample. Red represented a higher RPKM; blue represented a lower RPKM. Results were based on 2 RNA sequencing samples. (**B**) The volcano plot of differentially expressed genes. A *P* value of less than 0.05 and a fold change of more than 2 were set as restrictive conditions to identify the differentially expressed genes. (**C**) Pathway enrichment analysis showed differentially expressed genes which related to different pathways, among which the top-ranking enriched pathways were shown. (**D**–**F**) Identification of SRSF6-regulated alternative splicing events. (**D**) Number and proportion of different alternative splicing events modified in each category, as identified by rMATS analysis. (**E**) Skipped (green) and included (red) SRSF6-regulated cassette exons (CA exons) plotted by differential percent spliced (ΔPSI). (**F**) Venn diagram shows the overlap between differentially spliced genes and differentially expressed genes.

**Figure 8:**
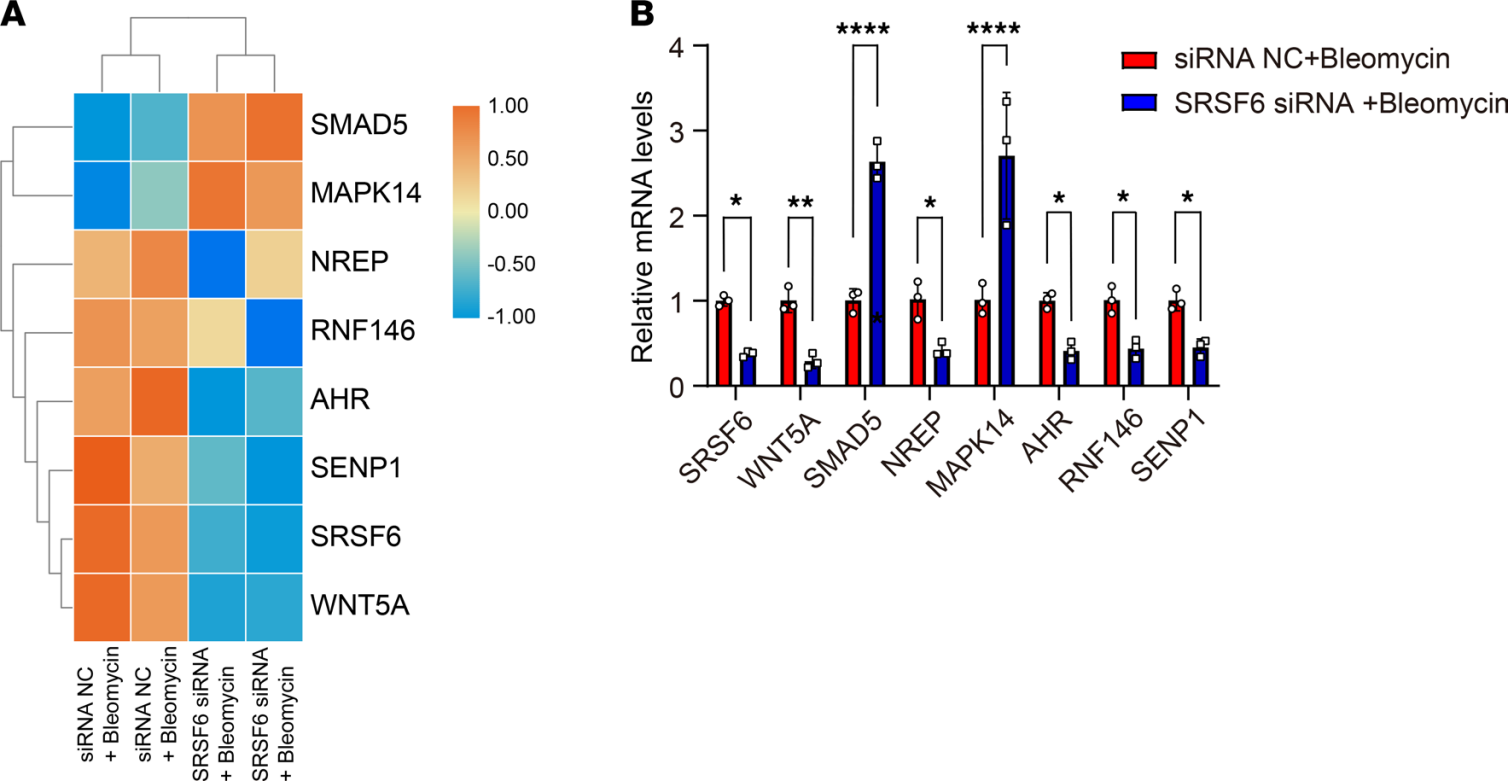
Confirmation of SRSF6 regulating mRNAs. (**A**) After transfection with human SRSF6 siRNA2 or negative control (NC) siRNA for 48 hours, PMCs were incubated with bleomycin (0.2 μg/mL) for 24 hours, after which whole RNA sequencing was performed. The heat map shows representative differentially expressed genes in bleomycin plus SRSF6 siRNA-treated cells compared with bleomycin-treated cells. (**B**) PMCs were treated as described for **A**, after which indicated mRNA levels were measured by RT-qPCR and normalized to the housekeeping gene GAPDH. Data are shown as mean ± SEM of *n* individual experiments. *n =* 3, *P < 0.05, **P < 0.01, ****P < 0.0001 (unpaired Student’s *t* test).

To explore downstream functional roles of SRSF6, RNA sequencing was performed in PMCs treated with bleomycin and SRSF6 siRNA or negative control siRNA (siRNA NC). There were 684 differentially expressed genes identified in cells treated by SRSF6 siRNA compared with siRNA NC, with 331 genes upregulated and 353 downregulated (Figure 7, A and B). Differentially expressed genes were enriched in pathways involved in RNA polymerase I chain elongation, TCF-dependent signaling in response to WNT, and signaling by WNT (Figure 7C). Interestingly, 5737 alternative splicing events were detected as modified (Figure 7D). Most of these modified alternative splicing events correspond to cassette exons (72%), followed by alternative 3′ splice site (11%), alternative 5′ splice site (7%), mutually exclusive exons (5%), and intron retention (5%). In cassette exons, 52.05% were exon skipping and 47.95% were exon inclusion changes (Figure 7E). This distribution of differential percent spliced in (ΔPSI) values in modified cassette exons suggested a possible dual role for SRSF6 as a splicing activator and repressor. We found 3164 differentially spliced genes. Notably, 93 differentially expressed genes also presented changes in alternative splicing (Figure 7F). We next used independent PMCs samples, different from those used for RNA sequencing, to confirm SRSF6-regulated differentially expressed genes. Representative genes from those known to regulate PMCs function were selected for further validation by RT-qPCR (Figure 8A). We validated 8 differentially expressed genes tested, indicating good reliability of the data generated by RNA sequencing (Figure 8B).

### Confirming the role of SRSF6/WNT5A and SRSF6/SMAD5 signaling in pleural fibrosis.

**Figure 9:**
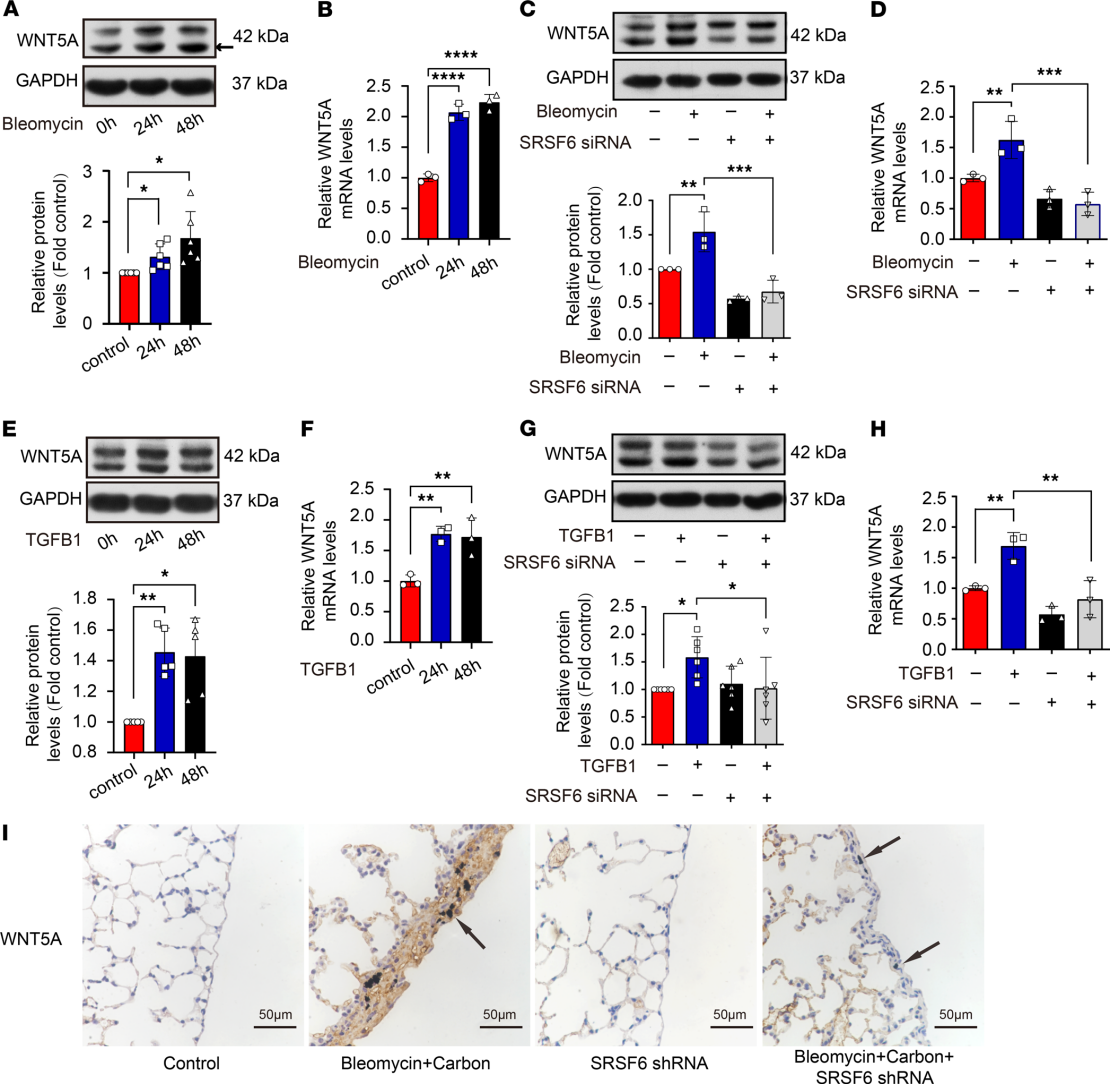
Bleomycin and TGF-β1–induced pleural fibrosis via SRSF6/WNT5A signaling pathway. (**A**–**H**) PMCs were incubated with bleomycin (0.2 μg/mL) or TGF-β1 (5 ng/mL) in the presence or absence of SRSF6 siRNA for 24 or 48 hours, after which intracellular WNT5A protein and mRNA levels were measured as described in Methods. (**A** and **E**) Representative Western blots and changes of density of WNT5A protein at 24 and 48 hours. (**A**) *n =* 6; (**E**) *n =* 5. (**B** and **F**) Changes of *WNT5A* mRNA at 24 and 48 hours, *n =* 3. (**C** and **G**) Representative Western blots and changes of density of WNT5A protein at 48 hours. (**C**) *n =* 3; (**G**) *n =* 6. (**D** and **H**) Changes of WNT5A mRNA, *n =* 3. Data are shown as mean ± SEM of *n* individual experiments. *P < 0.05, **P < 0.01, ***P < 0.001, ****P < 0.0001 (1-way ANOVA followed by the Dunnett’s test in **A**, **B**, **E**, and **F** or the Bonferroni’s test in **C**, **D**, **G**, and **H**). (**I**) Mouse pleural fibrosis model was induced by intrapleural injections of bleomycin plus carbon particles. IHC staining for WNT5A protein was performed on lung sections. Brown, WNT5A protein positive. Scale bar: 50 μm. Original magnification, ×400.

To confirm the role of SRSF6/WNT5A signaling in pleural fibrosis, SRSF6 siRNA and WNT5A siRNA were used to knock down expression in PMCs. As shown in Figure 9, A–H, treatment of PMCs with bleomycin or TGF-β1 increased WNT5A levels, whereas SRSF6 siRNA inhibited WNT5A expression. Furthermore, SRSF6 siRNA prevented bleomycin-induced WNT5A upregulation in vivo (Figure 9I).

To explore the role of WNT5A signaling in pleural fibrosis, several WNT5A siRNAs were designed and tested in PMCs (Supplemental Figure 13, A–C), WNT5A siRNA1 prevented bleomycin- or TGF-β1–induced COL1A2, ACTA2 synthesis, as well as cell proliferation (Supplemental Figure 13, D–I). These data provided evidence that SRSF6/ WNT5A signaling is involved in the mechanism of pleural fibrosis.

**Figure 10:**
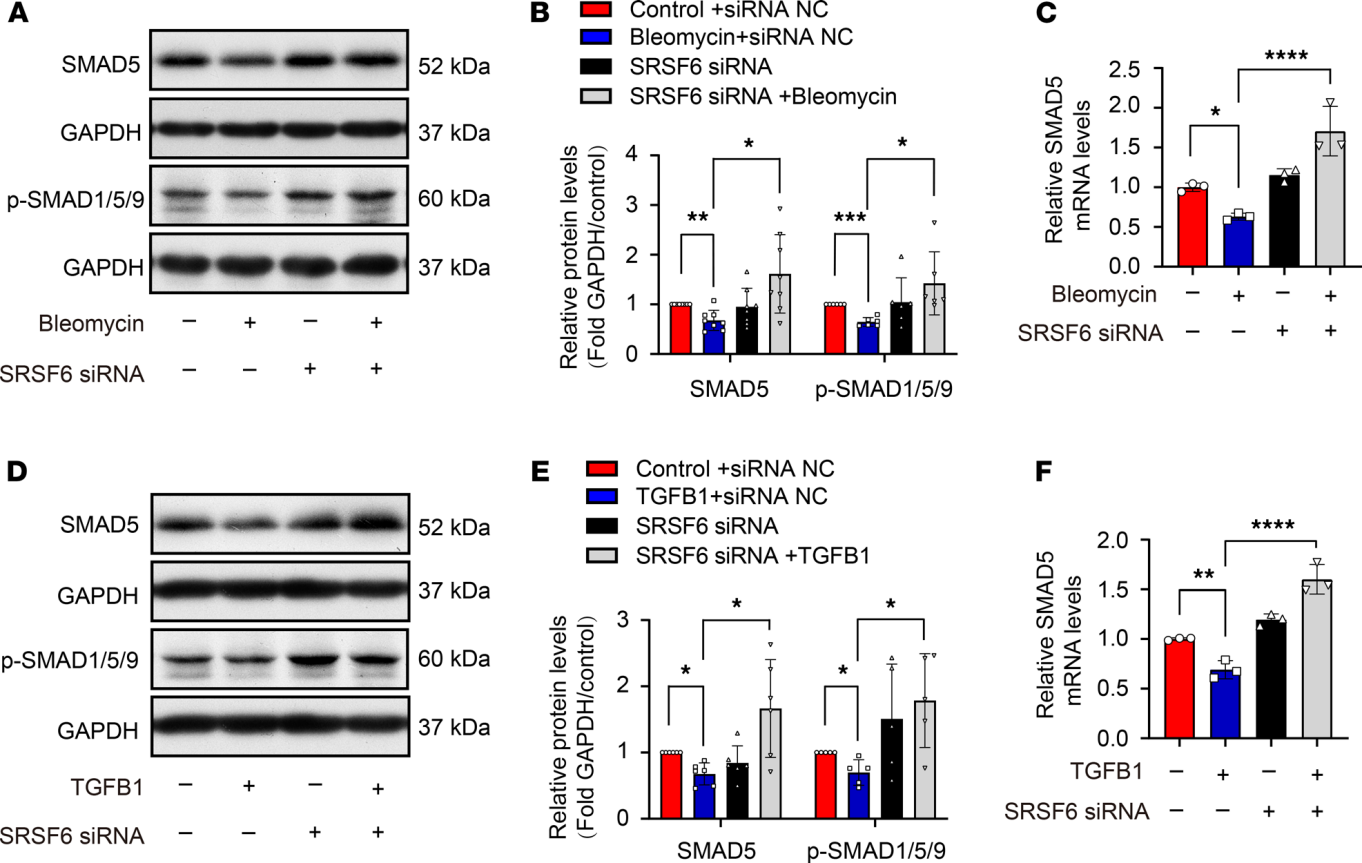
Bleomycin- and TGF-β1–induced pleural fibrosis via SRSF6/SMAD5 signaling. PMCs were incubated with bleomycin (0.2 μg/mL) or TGF-β1 (5 ng/mL) in the presence or absence of SRSF6 siRNA, after which intracellular SMAD5 protein and mRNA levels, and p-SMAD1/5/9 protein levels were measured as described in Methods. (**A** and **D**) Representative Western blots. (**B** and **E**) Changes of density of SMAD5 and p-SMAD1/5/9 protein. (**C** and **F**) Changes of *SMAD5* mRNA. Data are shown as mean ± SEM of *n* individual experiments. (**B**) *n =* 8 (SMAD5), *n =* 6 (p-smad1/5/9); (**C**) *n =* 3; (**E**) *n =* 6 (SMAD5), *n =* 5 (p-SMAD1/5/9); (**F**) = 3. *P < 0.05, **P < 0.01, ***P < 0.001, ****P < 0.0001 (1-way ANOVA followed by the Bonferroni’s test).

SMAD1/5/9 signaling is an antifibrotic pathway, which competes against SMAD2/3 signaling. Bleomycin and TGF-β1 treatment of PMCs inhibited SMAD5 mRNA and protein expression and depressed SMAD1/5/9 phosphorylation (Figure 10). However, these effects were restored by SRSF6 knock down. These data indicated that SRSF6/SMAD1/5/9 signaling is also involved in the mechanism of pleural fibrosis.

## Discussion

In this study, SRSF6 protein was found to be increased in cells of TBPE from patients. Next TBPE, bleomycin, and TGF-β1 were confirmed to increase SRSF6 levels in PMCs. More importantly, SRSF6 mediated expression of the main fibrotic protein COL1A2, as well as ACTA2 synthesis and PMC proliferation. In vivo, knock down of SRSF6 prevented mouse experimental pleural fibrosis. Finally, we investigated signaling upstream and downstream of SRSF6 in pleural fibrosis. We found activated SMAD2/3, increased SOX4, and depressed miR-506-3p mediated SRSF6 upregulation in PMCs. SRSF6 induced pleural fibrosis through a cluster pathway, including SRSF6/WNT5A and SRSF6/SMAD1/5/9 signaling.

In addition to serving as a protective barrier, the pleura are an immunologically and metabolically active membrane that is involved in maintaining homeostasis as well as in responding to pleural inflammation (10). As the first cells to recognize a perturbation in the pleural space, PMCs play a critical role in the initiation of inflammatory responses. In some cases, especially in tuberculous pleurisy patients, inflammatory responses result in pleural effusion, which is associated with dysfunctional wound repair as well as pleural fibrosis. Many kinds of cytokines are released into TBPE and likely take part in this pathophysiological process.

Bleomycin is a chemotherapeutic agent used in the treatment of a variety of tumors, including lymphoma, squamous cell carcinoma, and testicular carcinoma. However, chemotherapy using bleomycin is often complicated by interstitial pulmonary fibrosis (11, 12). Indeed, bleomycin has been widely used to generate animal models of pulmonary fibrosis (13).

TGF-β1 overproduction is the principal abnormality in most fibrotic diseases, and elevated levels of TGF-β1 have been found in pleural effusions (14). Intrapleural administration of TGF-β1 has been demonstrated to induce pleural fibrosis in animal models (15, 16).Therefore, in the current study, we treated PMCs with TBPE, bleomycin, and TGF-β1 to explore pleural fibrosis mechanisms.

It is now clear that most of the pre-mRNAs contain exons that can be alternatively included into the mature mRNA or removed from it, which is called alternative splicing. This process allows the production of multiple functionally distinct proteins from a single gene (17, 18). Alternative splicing is a crucial method for control of tissue-specific expression of related proteins. They constantly change under physiological conditions, allowing an organism to respond to changes in the environment by determining which part of the genome it expresses. However, alternative splicing can lead to human diseases (19). As a highly conserved RNA-binding splicing-factor protein, SRSF6 is essential for development and plays a role in cell growth (20). SRSF6 has also been implicated in cancer, skin hyperplasia, and diabetes (21–23). Kim and colleagues identified the related SRSF5 as a novel detection marker for pleural metastatic cancer cells (24). Manetti et al. reported that SRSF6 participated in fibrosis in patients with systemic sclerosis (9). In the current study, we provided evidence that SRSF6 mediates cell proliferation and COL1A2 synthesis in PMCs, and in vivo knock out of SRSF6 prevented pleural fibrosis in a bleomycin-induced mouse model. SRSF2 and SRSF5 did not change in PMCs treated by TGF-β1, bleomycin, or TBPE (Supplemental Figure 14). SRSF6 siRNA used in our experiments did not block other similar splicing factors (Supplemental Figure 15). This leads us to conclude that SRSF6 is a key mediator in pleural fibrosis.

Recently, SOX4 was confirmed to mediate cell survival and epithelial mesenchymal transition, and SOX4 activation depended on TGF-β/SMAD2/3 signaling (25). In PMCs, treatment with TBPE, bleomycin, or TGF-β1 activated SMAD2/3, which increased SOX4 levels. SOX4 then bound to the *SRSF6* promoter and induced expression in PMCs. miR-506-3p was found to downregulate SRSF6 expression through binding to the 3′UTR of the *SRSF6* mRNA. Interestingly, exosomal miR-506-3p levels were observed to be reduced in TBPE relative to TPE, suggesting that regulation of miR-506-3p expression also plays a role in SRSF6 regulation.

SRSF6 activated a cluster pathway, which should be involved in pleural fibrosis. Among the gene transcripts that were differentially spliced in PMCs with SRSF6 knock down, WNT5A and SMAD5 were selected for further study because of their known signaling roles. Aberrant WNT5A signaling is associated with several human pathologies such as inflammation, fibrosis, and cancer. Fibroblasts from pulmonary fibrosis patients and keloid regions show increased proliferation, survival, and ECM protein expression (26, 27). WNT5A mRNA and protein expression is increased in these fibroblasts. WNT5A predominantly activates β-catenin–independent WNT signaling and can also activate β-catenin signaling to influence diverse cellular effects such as cell migration, proliferation, and resistance to apoptosis (28). Thus, WNT5A promotes proliferation and survival of lung fibroblasts, and augments fibronectin and integrin expression. Similarly, in the current study, WNT5A mRNA and proteins increased in PMCs in vitro and in vivo. SRSF6 knock down prevented WNT5A activation as well as pleural fibrosis.

The TGF-β signaling pathway plays a key role in fibrosis and can be mediated by the SMAD family of proteins. SMAD2 and SMAD3 are substrates for receptors triggered by TGF-βs and activins. SMAD1, SMAD5, and SMAD9 mediate the pathways stimulated by BMPs, GDFs, and MIFs. The TGF-βRII/ALK5 complex activates SMAD2 and SMAD3, but the TGF-βRII/ALK1 complex activates SMAD1, SMAD5, and SMAD9 (29). The ALK1/SMAD1/5/9 pathway is an antagonistic mediator of ALK5/SMAD2/3 signaling (30), and owing to this antagonism, it regulates negatively ECM protein expression in cells. In our study, bleomycin and TGF-β1 activated SMAD2/3/SOX4 signaling, which upregulated SRSF6 in PMCs. Moreover, we verified that increased SRSF6 inhibited SMAD5 and p-SMAD1/5/9, which also played a role in pleural fibrosis.

In conclusion, SRSF6 protein increased in pleural fibrosis. SMAD2/3/SOX4 and miR-506-3p regulated SRSF6 expression, and increased SRSF6 mediated pleural fibrosis through a cluster pathway, including SRSF6/WNT5A and SRSF6/SMAD5 signaling.

## Methods

### Reagents and Abs.

Recombinant human TGF-β1 and TGF-β1 receptor inhibitor (SB431542) were purchased from R&D Systems. Carbon particles were obtained from Mitsubishi Chemical Corporation. Bleomycin was obtained from Tianjin Taihe Pharmaceutical Co., Ltd. Abs used were as follows: anti-SRSF6 (PA5-41810, Invitrogen and sc-57954, Santa Cruz Biotechnology), anti-COL1A2 (14695-1-AP, Proteintech), anti-ACTA2 (14395-1-AP, Proteintech), anti-SOX4 (B-7) (sc-518016, Santa Cruz Biotechnology), anti-WNT5A (abs113167, Absin), anti-TGF-β1 (21898-1-AP, Proteintech), anti-SMAD5 (12167-1-AP, Proteintech), anti-phospho-SMAD1/5/9 (P-SMAD1/5/9) (13820, Cell Signaling Technology), anti-SMAD2/3 (8685, Cell Signaling Technology), anti-phospho-SMAD2/3 (p-SMAD2/3) (8828, Cell Signaling Technology), anti-GFP (2956, Cell Signaling Technology), anti-SRSF5 (16237-1-AP, Proteintech), anti-GAPDH (60004-1, Proteintech), and IgG (10283-1-AP, Proteintech).

### Cell culture.

The human PMC line Met-5A was obtained from the ATCC. PMCs were cultured in RPMI 1640 medium (Gibco) supplemented with 10% FBS. HEK293T cells were cultured in high-glucose DMEM medium (Gibco) supplemented with 10% FBS. The mouse lung epithelial cell line MLE-12 was obtained from ATCC and cultured in RPMI 1640 medium supplemented with 10% FBS. All culture media contained 100 U/mL penicillin and 100 μg/mL streptomycin, and cell cultures were maintained at 37°C in a humidified atmosphere containing 5% CO_2_. Cells were subcultured at 1:3 ratios, and culture medium was changed every 2 days. Cells equilibrated overnight in medium containing 2% FBS were used for all experiments.

### Pleural effusion samples collection and processing.

TBPE criteria included the identification of *Mycobacterium tuberculosis* in pleural fluid or the demonstration of granulomatous pleurisy in closed pleural biopsy specimen in the absence of any evidence of other granulomatous diseases. TPE was determined according to Light’s criteria. A total of 100–500 mL TBPE or TPE samples from each patient was collected in heparin-treated tubes, through a standard thoracocentesis technique within 24 hours after hospitalization. TBPE or TPE specimens were immersed in ice immediately and then centrifuged at 1000*g* for 10 minutes at 4°C. Supernatants were aliquoted and stored at –80°C for experiments. For detailed patient information, please see Supplemental Table 1.

### Exosome isolation from pleural effusion and miRNA-Seq.

TBPE or TPE specimens were centrifuged at 300*g* for 10 minutes at 4°C. The supernatant was taken and centrifuged at 10,000*g* for 30 minutes at 4°C. Then, the supernatant was ultracentrifuged at 100,000*g* for 70 minutes at 4°C. The pellet was resuspended in PBS buffer and ultracentrifuged again at 100,000*g* for 70 minutes. After ultracentrifugation, the pellet was suspended with 50 μL PBS buffer and saved at –80°C. Total RNA in exosome was isolated from 3 independent preparations using exoRNeasy Serum/Plasma Maxi Kit (catalog 77023, Qiagen) according to the manufacturer’s protocol. miRNA-Seq was performed by Novogene Co., Ltd. on an Illumina HiSeq 2500 sequencer. The raw data were deposited in the Gene Expression Omnibus (GEO) database (GSE172150).

### Isolation of human primary PMCs from pleural effusion.

Human primary PMCs were isolated from pleura effusion as described in our previous study (31). In brief, the fresh pleural effusion was filtered with gauze to remove impurities and then centrifuged at 1000*g* for 10 minutes at 4°C. The precipitated cells were washed with PBS and then centrifuged at 1000*g* for 5 minutes at 4°C. After being washed twice with PBS, the cells were used to extract protein and mRNA.

### Western blot assay for total cell lysates.

Western blotting was performed according to standard method. The proteins of interest were probed with primary Abs (listed as “target protein” [dilution factor]): SRSF6 (1:1000), COL1A2 (1:1000), ACTA2 (1:2000), SOX4 (1:500), WNT5A (1:500), SMAD5 (1:1000), p-SMAD1/5/9 (1:500), SMAD2/3 (1:1000), p-SMAD2/3 (1:500), GAPDH (1:10000), and SRSF5 (1:1000).

### RNA extraction and qRT-PCR.

After treatment, total RNA was extracted using TRIzol reagent (Invitrogen) according to the manufacturer’s protocol. qRT-PCR was performed under the following conditions: 95°C for 30 seconds, 40 cycles of 95°C for 10 seconds, and 55°C to 60°C for 30 seconds. Target gene expression levels were normalized to GAPDH expression and then calculated using the 2^-ΔΔCt^ method. The primer sequences are listed in Supplemental Table 2.

### Immunofluorescence staining.

To determine the intracellular localization and changes in SRSF6, COL1A2, and ACTA2, PMCs were incubated with bleomycin (0.2 μg/mL), TGF-β1 (5 ng/mL), and TBPE (5%) for 48 hours. Then, the cells were stained using Abs against SRSF6 (dilution 1:200), COL1A2 (dilution 1:100), or ACTA2 (dilution 1:200) at 4°C overnight and then with FITC-labeled secondary Ab IgG (SA00003, Proteintech) for 1 hour. The nuclei in PMCs were stained for DAPI for 6 minutes in the dark. For mouse tissues, specific tissues were fixed in 4% paraformaldehyde in PBS for 48 hours.

### Cell proliferation assay.

Cell proliferation was measured by CCK-8 and EdU assays. PMCs grew in 96-well plates after initial seeding at a density of 3 × 10^3^ cells per well. Proliferation of cells was assayed using Cell Counting Kit-8 (Dojindo) according to the manufacturer’s instruction. For the EdU assay, proliferation of cells was measured by Baseclick EdU Cell Proliferation Kit (BCK-EDU555, Sigma-Aldrich) according to the manufacturer’s instructions.

### Bleomycin-induced pleural fibrosis model and treatments.

The mouse pleural fibrosis model was induced by bleomycin as described previously by us (31) and described in detail as follows. C57BL/6J mice (males, 6–8 weeks of age, 18–20 g) were maintained under specific pathogen-free conditions. A mixture of bleomycin (0.48 μg/mouse) and carbon particles (0.1 mg/mouse) was administered in a volume of 100 μL saline solution by intrapleural injection on day 1. For gene knock down, shRNA fragments of targeting mouse SRSF6 were cloned into the plasmid pGCL-GFP, which encodes an HIV-derived lentiviral vector containing a multiple cloning site for insertion of shRNA construct to be driven by an upstream U6 promoter and a downstream cytomegalovirus promoter-GFP (marker) cassette flanked by loxP sites. Three shRNA sequences were pretested for mouse SRSF6, and the shRNA3 (5′-cgTACAGAGTACAGGCTTATT-3′) was selected as the most efficient for later studies. All lentiviruses were constructed by GeneChem. Lentiviral vector containing nontargeting control shRNA (5′-TTCTCCGAACGTGTCACGT-3′) was constructed as negative control. Mice in the control group received intrapleural injection with 100 μL saline solution. Lentivirus expressing shRNA directed against SRSF6 or scrambled sequence shRNA were administrated by intrapleural injection at a dose of 2 × 10^6^ TU on days 4, 7, and 10. All mice were euthanized after 21 days, and the tissues were taken for histological analysis.

### Histological analysis.

The specific mouse tissue samples were fixed in 4% paraformaldehyde for 48 hours and then were sliced midsagittally and embedded in paraffin. For morphological analysis and evaluation of collagen deposition, standard Masson staining and Sirius red staining were performed on 5 μm thick tissue sections as previously described (32). The slides were examined by using a microscope connected with a digital camera, and Image-Pro Plus software was used to analyze the images.

### Immunohistochemical staining.

Paraffin-embedded sections of mice tissue (5 μm thick) were used for IHC. Samples were deparaffinized and rehydrated, followed by antigen retrieval by boiling Citrate Antigen Retrieval Solution (G1201, Servicebio) for15 minutes. Standard IHC was performed using UltraSensitive SP IHC Kit (KIT-9710, MXB Biotechnologies) according to the manufacturer’s instructions. Sample sections were exposed to primary Abs, including anti-COL1A2 (1:2000), anti-ACTA2 (1:1500), anti-WNT5A (1:1000), anti-SRSF6 (1:100, sc-57954; Santa Cruz Biotechnology), anti-TGF-β1 (1:1500), and anti-SOX4 (1:100) at 4°C overnight, and then incubated with biotinylated secondary Abs for 20 minutes. The slides were developed with DAB working solution, followed by counterstaining with hematoxylin and mounting with mounting medium. The slides were scanned using a microscope connected with a digital camera, and Image-Pro Plus software was used to analyze the images.

### Gene silencing.

We silenced specific gene expression in PMCs by using siRNA-mediated silencing. Transient transfection was performed using 50 nmol/L siRNA and Lipofectamine RNAiMAX (Invitrogen) in serum-free medium according to the manufacturer’s instructions. All siRNAs were obtained from GenePharma. The sequences of siRNAs targeting specific genes are described in Supplemental Table 2.

### ChIP assay.

ChIP assays were performed as previously described (33). PMCs were subjected to 1% formaldehyde incubation for 8 minutes. The cells were collected and lysed by sonication to obtain chromatin fragments of 200–300 bp. Untreated sheared chromatin was used as input. Then, cell lysates were immunoprecipitated with anti-SOX4 (B-7) (sc-518016; Santa Cruz Biotechnology) or IgG (10283-1-AP; Proteintech) as a control. After washing and elution, cross-links were reversed for 4 hours at 65°C. The eluted DNA was purified by DNA Purification Kit (D0033, Beyotime). Purified DNA was analyzed by PCR (P213, Vazyme) and qPCR (Q311, Vazyme) with the primers described in Supplemental Table 2.

### Quantification of miRNA.

Total cellular RNA was extracted using TRIzol reagent (Invitrogen). miR-cDNAs were synthesized from total RNA by reverse transcription using a One Step PrimeScript miRNA cDNA Synthesis Kit (Takara). Real-time PCR was performed on the Quantagene q225 (Kubo Technology) using Cham Q SYBR qPCR Master Mix (Q311, Vazyme) and with U6 snRNA as the internal control. miR-506-3p and U6 snRNA primers were obtained from Integrated Biotech Solutions.

### miR-506-3p overexpression and inhibition.

miR-506-3p unique RNA-based mimics, inhibitors, and NC nontargeting oligonucleotides were synthesized by GenePharma. miRNA mimics (50 nM/L) and the mimic NC (50 nM/L) were transfected into PMCs using Lipofectamine RNAiMAX (Invitrogen) according to the manufacturer’s protocol. miRNA inhibitors (10 nM/L) and the NC were transfected using Lipofectamine RNAiMAX transfection reagent (Invitrogen) for 48 hours. The sequences of miRNA mimics and inhibitors are listed in Supplemental Table 3.

### Dual-luciferase reporter assay.

The 232 nt sequence of the WT *SRSF6* (NM_006275.6) 3′ UTR containing the putative seed binding sequence for miR-506-3p (ACGGAAU) was synthesized and subcloned into pMIR-Report luciferase. A control was generated with a mutation of the miR-506-3p binding sequence in the human *SRSF6* 3′ UTR that changed the seed binding sequence to GAUUGCG. We first established stably overexpressed miR-506-3p cell line by transducing HEK293T cells with mimics expressing miR-506-3p and NC. A total of 2 × 10^5^ cells/well were then seeded in a 24-multiwell plate, and plasmids were transfected the following day using lipofectamine 2000 (Invitrogen) according to the manufacturer’s instructions. Cells were harvested after 24 hours. Firefly and renilla luciferase were determined using the dual-luciferase reporter assay system (Promega) with a Synergy 2 Multi-Mode microplate reader (BioTek Instruments). Relative luciferase signals were standardized according to firefly/renilla ratio.

### RNA-Seq and analysis.

After transfection with SRSF6 siRNA or siRNA NC, PMCs were incubated with bleomycin (0.2 μg/mL) for 24 hours, after which total RNA was isolated from 2 independent preparations using TRIzol reagent (Invitrogen), according to the manufacturer’s protocol. RNA-Seq was performed by CapitalBio Technology on an Illumina NovaSeq sequencer (Illumina). The raw data were deposited in GEO under submission GSE168578. For analysis, Clean reads were aligned to GRCh38 using HISAT2 (v2.1.0; ref. 34) with default parameters. Gene expression analyses were carried out with StringTie (v1.3.3b; ref. 35). DESeq (v1.28.0; ref. 36) was used to analyze the DEGs between samples. Splicing events were identified by ASprofile (37). Alternative isoform was analyzed by rMATS (38–40). The splicing efficiency was quantified by inclusion level (PSI, percentage spliced in) and statistically analyzed by likelihood-ratio test. The splicing events with FDR < 0.01 and | IncLevel1 –IncLevel2 | > 0.05 were considered statistically significant.

### Statistics.

Potential SOX4 binding sites of *SRSF6* were analyzed with the JASPAR database (http://jaspar.genereg.net). The potential mRNA targets of miR-506-3p were analyzed with the TargetScan database (http://www.targetscan.org). For statistical analysis, data analyses were performed using GraphPad Prism 8 software. Results are shown as the mean ± SEM of individual experiments. Control and experimental cells were matched for cell line, age, seeding density, number of passages, and number of days after confluence. Data are expressed as mean ± SEM of *n* individual experiments. In clinical samples and animal experiments, *n* means *n* samples from *n* different patients, animals, or control subjects. In cellular experiments, *n* means *n* samples from *n* individual experiments of cells, which cultured in individual dishes. Statistical significance between groups were determined by 2-tailed Student’s *t* test or 1-way or 2-way ANOVA. *P* values of less than 0.05 were considered significant.

### Study approval.

Experiments with human participants were approved by the IRB of Union Hospital, Tongji Medical College, Huazhong University of Science and Technology (2020-IEC-J-187). Written informed consent was obtained from all subjects. All animal experiments were performed in accordance with the *Guide for the Care and Use of Laboratory Animals* (National Academies Press, 2011) and were approved by the IACUC of Tongji Medical College, Huazhong University of Science and Technology (2020-S411).

## Author contributions

LML, HY, and WLM conceived the study. LML, LX, and HY designed the experiments. LML, LX, PPC, SJC, XLH, YYZ, and QN performed the experiments. MW, QC, LJS, FY, XLH, FX, and XW analyzed the data. LML, HY, and WLM prepared the figures and wrote the manuscript with input from all authors. Co-first authors were listed based on the order in which they began working on the project.

## Supplementary Material

Supplemental data

## Version 1 04/27/2021

In-Press Preview

## Version 2 05/24/2021

Electronic publication
