# Supplementary material for: Splicing factor SRSF6 mediates pleural fibrosis
Source: JCI Insight. 2021 May 24;6(10):e146197. doi: 10.1172/jci.insight.146197 (PMC8262297; doi:10.1172/jci.insight.146197)
Supplement: Supplemental data [file jciinsight-6-146197-s219.pdf]

Supplementary data

Title: Splicing factor SRSF6 mediates pleural fibrosis

Authors: Li-Mei Liang, Liang Xiong, Pei-Pei Cheng, Shuai-Jun Chen, Xiao Feng, Ya-Ya Zhou, Qian Niu, Meng Wang, Qianlan Chen, Lin-Jie Song, Fan Yu, Xin-Liang He, Fei Xiang, Xiaorong Wang, Hong Ye, Wan-Li Ma

Contents:

Supplemental Methods:

1. Isolation and culture of rat primary PMCs.
2. *In situ* hybridization histochemistry.

Supplemental Figure 1. SRSF6 protein expression in tuberculous pleurisy tissue.

Supplemental Figure 2. SRSF6 and calretinin expression in tuberculous pleurisy tissue.

Supplemental Figure 3. Bleomycin and TGFB1 increased SRSF6 and COL1A2 protein in PMCs.

Supplemental Figure 4. SRSF6 knockdown prevented TBPE-, bleomycin- and TGFB1-induced proliferation *in vitro*.

Supplemental Figure 5. SRSF6 knockdown by siRNA suppressed TBPE-, bleomycin- and TGFB1-induced COL1A2 and ACTA2 synthesis *in vitro*.

Supplemental Figure 6. SRSF6 knockdown by siRNA suppressed TBPE-induced COL1A2 and ACTA2 synthesis in rat primary PMCs.

Supplemental Figure 7. SRSF6 shRNA transfection in mouse pleural fibrosis model.

Supplemental Figure 8. SRSF6 knockdown by shRNA attenuated bleomycin induced pleural fibrosis in mouse model.

Supplemental Figure 9. SOX4 siRNA attenuated SRSF6 expression in PMCs.

Supplemental Figure 10. SRSF6, TGFB1 and SOX4 protein changes in mouse pleural fibrosis model.

Supplemental Figure 11. Overexpression of miR-506-3p prevented TBPE-, bleomycin- and TGFB1-induced SRSF6, COL1A2 and ACTA2 synthesis in PMCs.

Supplemental Figure 12. Overexpression of miR-506-3p prevented TBPE-, bleomycin- and TGFB1-induced PMC proliferation.

Supplemental Figure 13. Bleomycin- and TGFB1-induced COL1A2 synthesis and cell proliferation via activation of WNT5A in PMCs.

Supplemental Figure 14. Bleomycin did not change SRSF2 or SRSF5 in PMCs.

Supplemental Figure 15. SRSF6 siRNA did not block siRNA expression of SRSF2, SRSF4, SRSF5 or SRSF7 in PMCs.

Supplemental Table 1. Clinical characteristics of patients with tuberculous pleural effusion (TBPE) and transudative pleural effusion (TPE)

Supplemental Table 2. Sequences of RT-PCR/qPCR primers and siRNA oligonucleotides

Supplemental Table 3. miRNA sequences for hsa-miR-506-3p

Supplemental Methods

1. Isolation and primary culture of rat primary PMCs. Rat primary PMCs were isolated from rat pleura with protease as our previous studies (1, 2). In brief, the whole thorax was isolated under sterile conditions after 1 mg/ml protease from streptomyces griseus (Sigma) in 5 ml RPMI-1640 medium injected into thoracic cavity and then digested at 4 °C overnight. The cells were harvested and centrifuged at 1, 000 rpm for 5 minutes (min). The spun down cells were resuspended in epithelial cell medium-animal (ScienCell, Carlsbad, CA, USA) and incubated for 7 days and then replaced with RPMI-1640 containing 20% FBS. Other treatments were the same as with Met-5A cells.

2. In situ hybridization histochemistry. To investigate *SRSF6* mRNA levels in the mouse pleura, in situ hybridization histochemistry was performed on the pleura tissues. The slides were incubated with hybridization buffer containing 5′-ATGCA GATGC TCACA AAGAA CGAAC AAATG AGGGT GTGAT-3′; 5′-ACGCC CGGGG CCCGC GCCGC GACCG CGATG GCTAC AGCTA-3′; 5′-GTGCT TTGGA TAAAC TGGAT GGTAC AGAAA TAAAT GGCAG-3′ probe (BOSTER, Wuhan, China) at 42 °C overnight. After added the blocking buffer, the slides were incubated with biotin-conjugated IgG fraction mouse anti-digoxin antibody, and subjected to streptavidin-biotin complex (SABC). Finally, the slides were undergoing chromogenic reaction with DAB kit.

References:

1. Song LJ, et al. Lethal (2) giant larvae regulates pleural mesothelial cell polarity in pleural fibrosis. *Biochim Biophys Acta Mol Cell Res.* 2018;1865(9):1201-1210.

2. Yang J, et al. Activation of calpain by renin-angiotensin system in pleural mesothelial cells mediates tuberculous pleural fibrosis. *Am J Physiol Lung Cell Mol Physiol*. 2016;311(1):L145-5

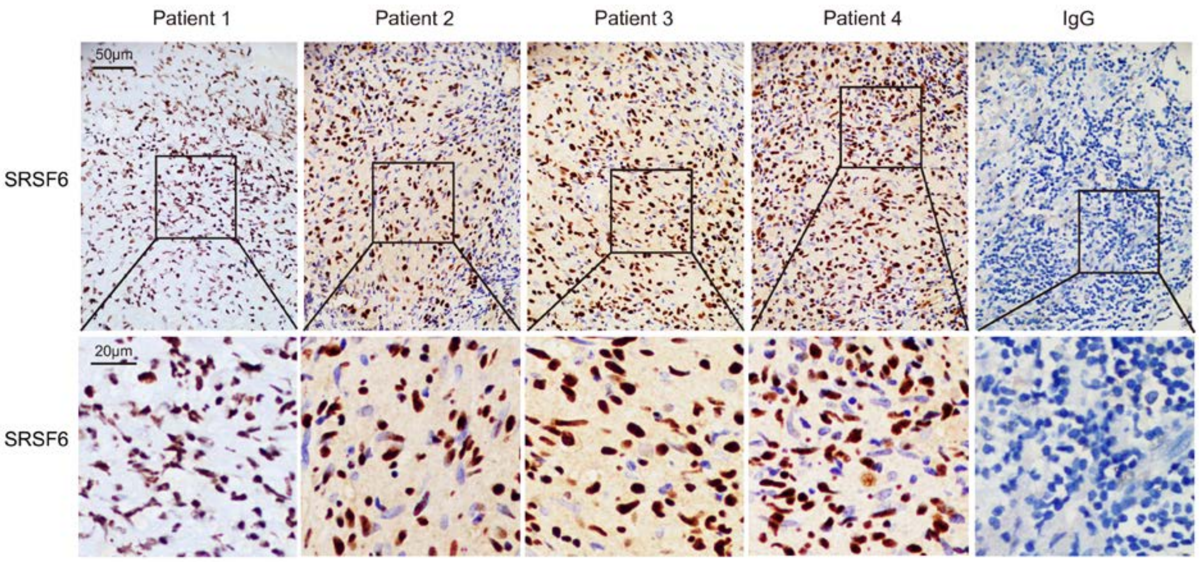

Supplemental Figure 1. SRSF6 protein expression in tuberculous pleurisy tissue. Pleural tissue samples were collected from parietal pleura in patients with tuberculous pleural effusion. Pleura sections were stained with immunohistochemical (IHC) staining using antibody against human SRSF6. Brown color shows SRSF6. Blue color shows nuclei. Original magnification, ×400.

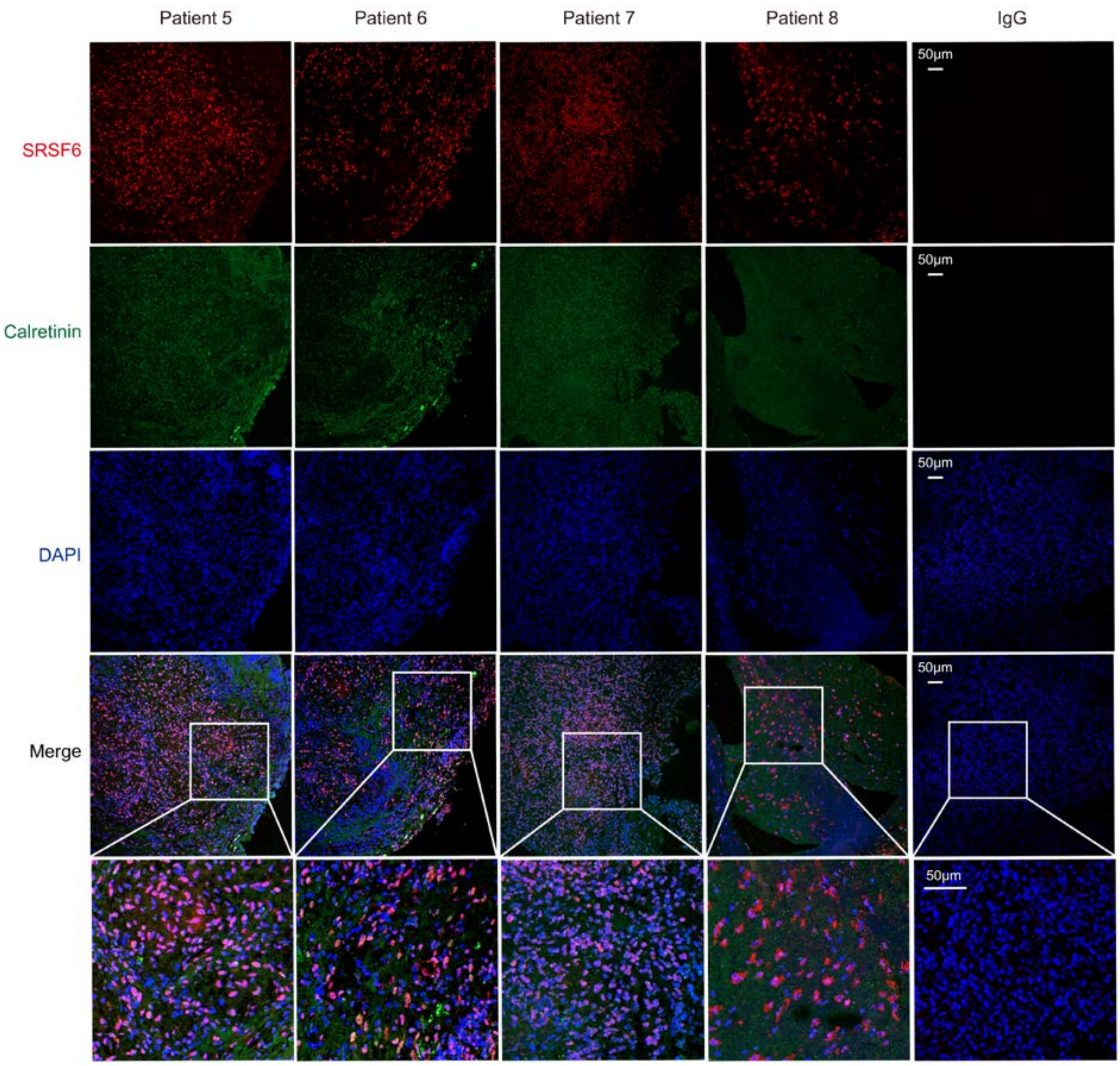

Supplemental Figure 2. SRSF6 and calretinin expression in tuberculous pleurisy tissue. Pleural tissue samples were collected from parietal pleura in patients with tuberculous pleural effusion. Pleura sections were stained with immunofluorescence (IF) staining using antibody against human SRSF6 and calretinin (a maker of pleural mesothelial cells). Red color shows SRSF6. Green color shows calretinin. Blue color showed nuclei (DAPI). Original magnification, ×100.

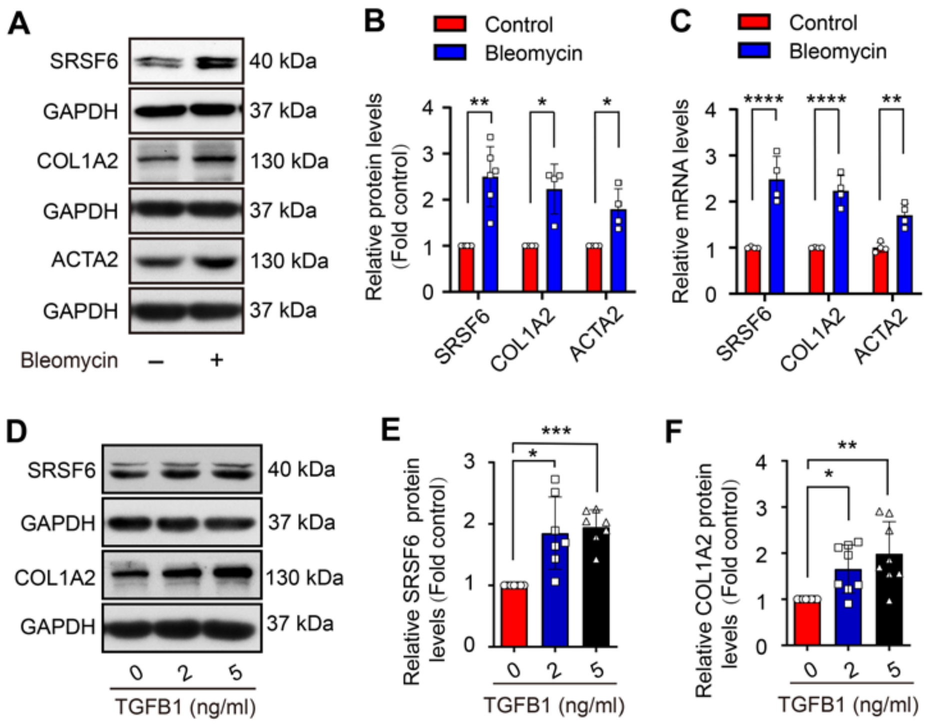

Supplemental Figure 3. Bleomycin and TGFB1 increased SRSF6 and COL1A2 protein in PMCs. Human PMCs were incubated with bleomycin (0.2 µg/ml) or TGFB1 (2, 5 ng/ml) for 48 h, after which intracellular protein levels of SRSF6, COL1A2 and ACTA2 were measured by western blotting. Representative western blots, and changes in relative density of SRSF6, COL1A2 or ACTA2 to GAPDH were presented. mRNA levels were detected by qRT-PCR and normalized by the housekeeping gene GAPDH. Data are expressed as mean ± SEM of n individual experiments. P values were determined by unpaired student's t-test (B, C) or one-way ANOVA followed by the Dunnett's test (E, F). B and C: n=6 (SRSF6), n=4 (COL1A2 and ACTA2); E: n=7; F: n=8. **P*<0.05, ***P*<0.01, ****P*<0.001 versus control group.

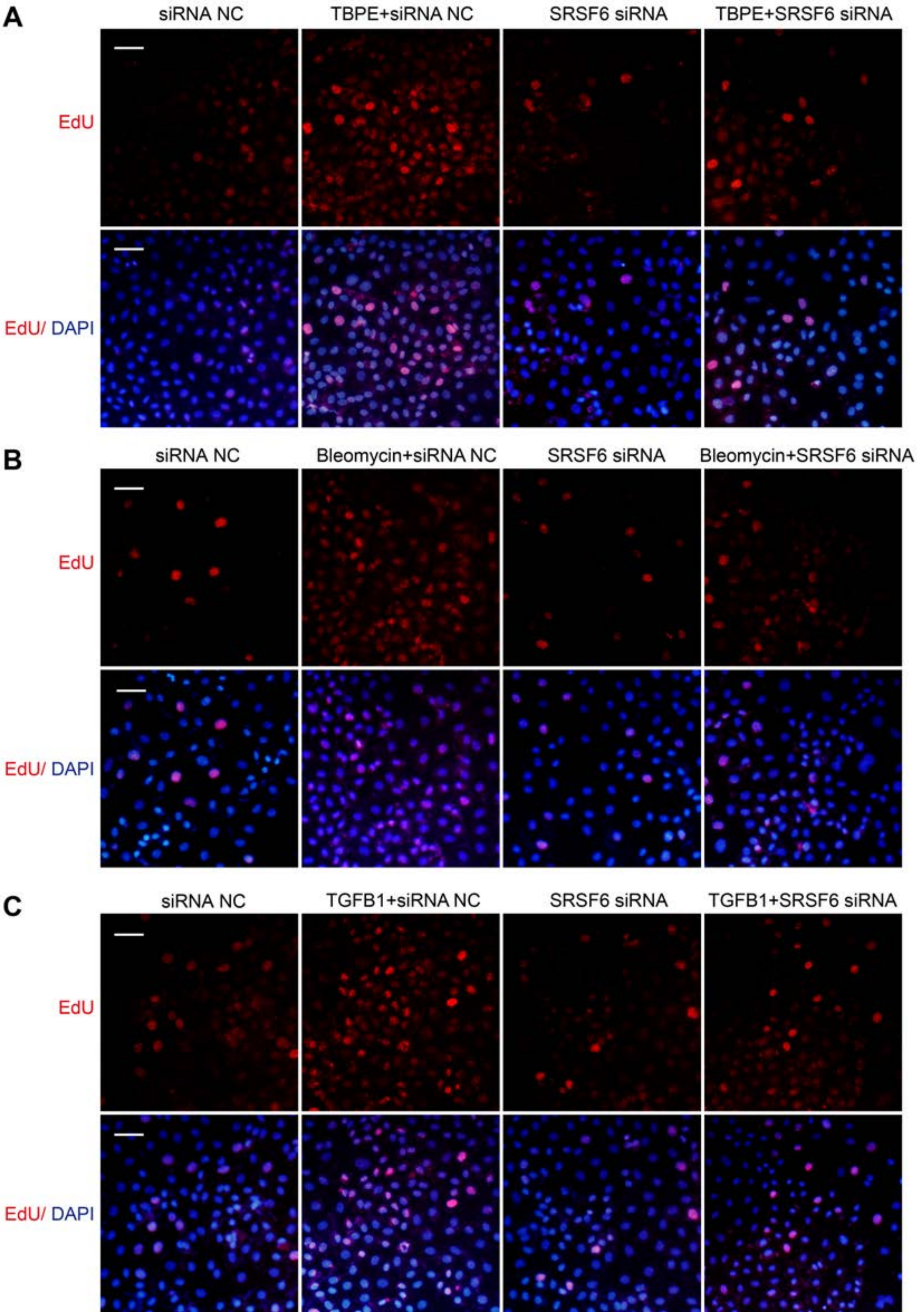

Supplemental Figure 4. SRSF6 knockdown prevented TBPE, bleomycin and TGFB1-induced proliferation *in vitro*. After transfection with SRSF6 siRNA or negative control siRNA (siRNA NC), PMCs were incubated with or without TBPE (5%), bleomycin (0.2 μg/ml) or TGFB1 (5 ng/ml) for 24 h, and cell proliferation was determined by EdU immunofluorescence staining. Red: EdU; Blue: DAPI. Scale bar: 100 μm. Original magnification, ×200.

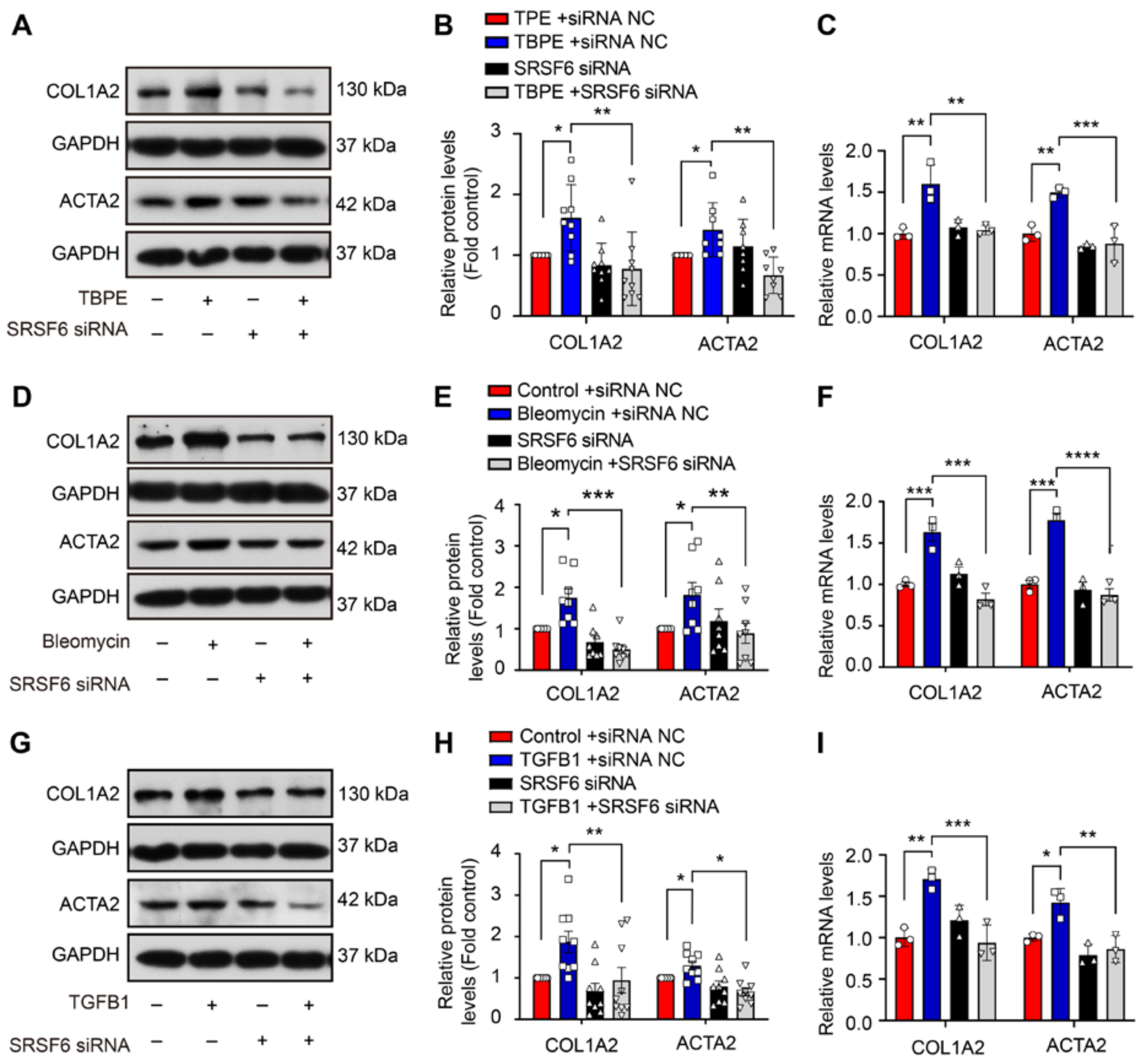

Supplemental Figure 5. SRSF6 knockdown by siRNA suppressed TBPE-, bleomycin- and TGFB1-induced COL1A2 and ACTA2 synthesis *in vitro*. After transfection with human SRSF6 siRNA or negative control (NC) siRNA, PMCs were incubated with or without TBPE (5%), bleomycin (0.2 μg/ml), or TGFB1 (5 ng/ml) for 48 h, after which intracellular COL1A2 and ACTA2 protein was measured by western blotting (A, D, G). mRNA levels were detected by RT-qPCR and normalized by the housekeeping gene GAPDH (C, F, I). Data are expressed as mean ± SEM of n individual experiments, B: n=9 (COL1A2), n=8 (ACTA2); C: n=3; E: n=8; F: n=3; H: n=9; I: n=3. *$P<0.05$, **$P<0.01$, ***$P<0.001$, ****$P<0.0001$ (One-way ANOVA followed by the Bonferroni's test).

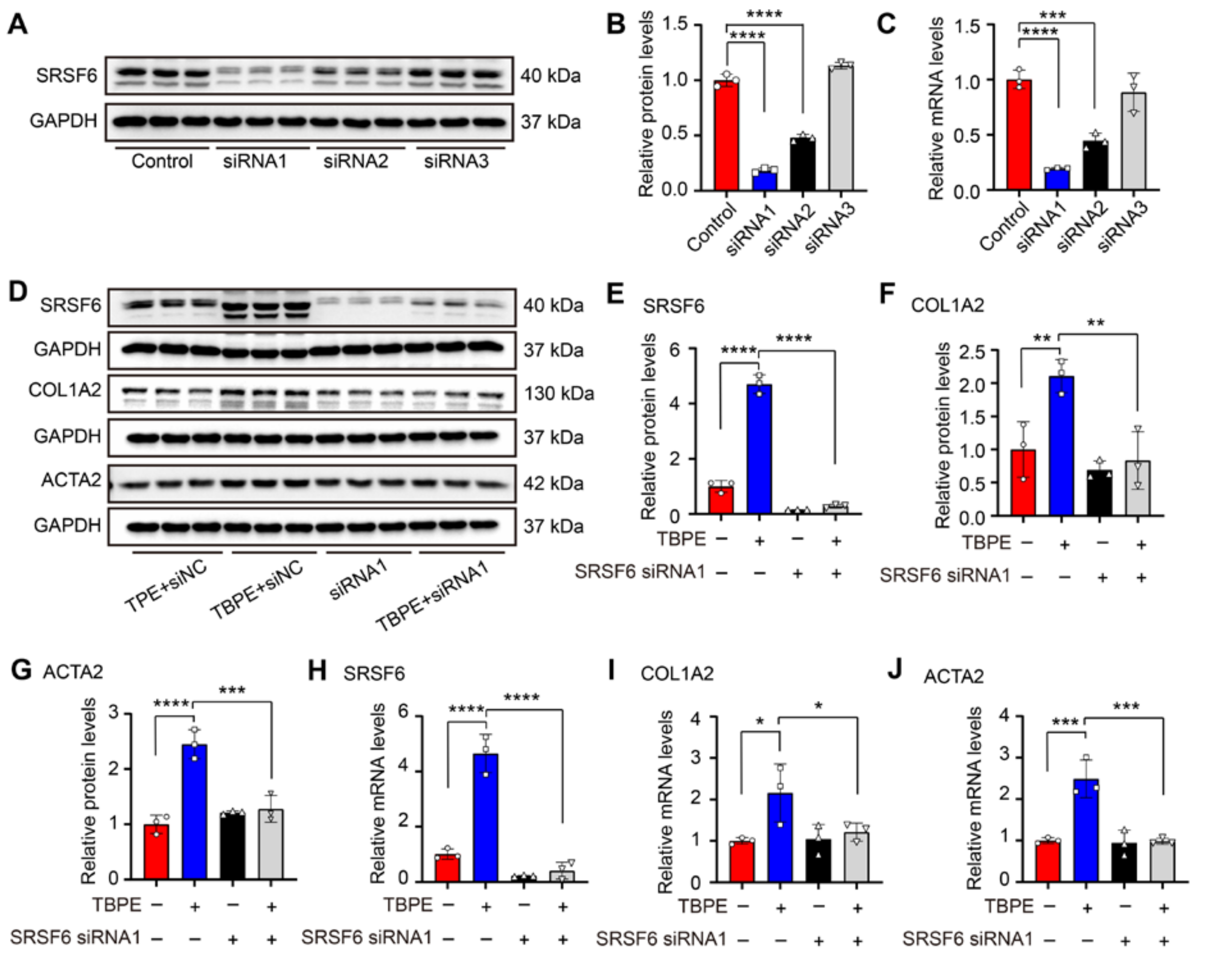

Supplemental Figure 6. SRSF6 siRNA suppressed TBPE-induced COL1A2 and ACTA2 synthesis in rat primary PMCs. (A-C) Rat primary PMCs were transfected with control or specific siRNA against SRSF6 (siRNA1, siRNA2 or siRNA3) for 48 h. SRSF6 protein and mRNA levels were assessed by western blotting (A, B), and qRT-PCR (C). Data are expressed as mean ± SEM of n individual experiments. n=3 (B), n= 3 (C), ****P*< 0.001, *****P*<0.0001 versus control (Student's t-test). (D-J) After transfection with SRSF6 siRNA1 (siRNA1) or negative control siRNA (NC siRNA), PMCs were incubated with or without TBPE (5%) for 24 h, protein and mRNA levels were assessed by western blotting (D-G), and RT-qPCR (H-J) Data are expressed as mean ± SEM of n individual experiments, n= 3. **P*<0.05, ****P*<0.001, *****P*<0.0001 (One-way ANOVA followed by the Bonferroni's test).

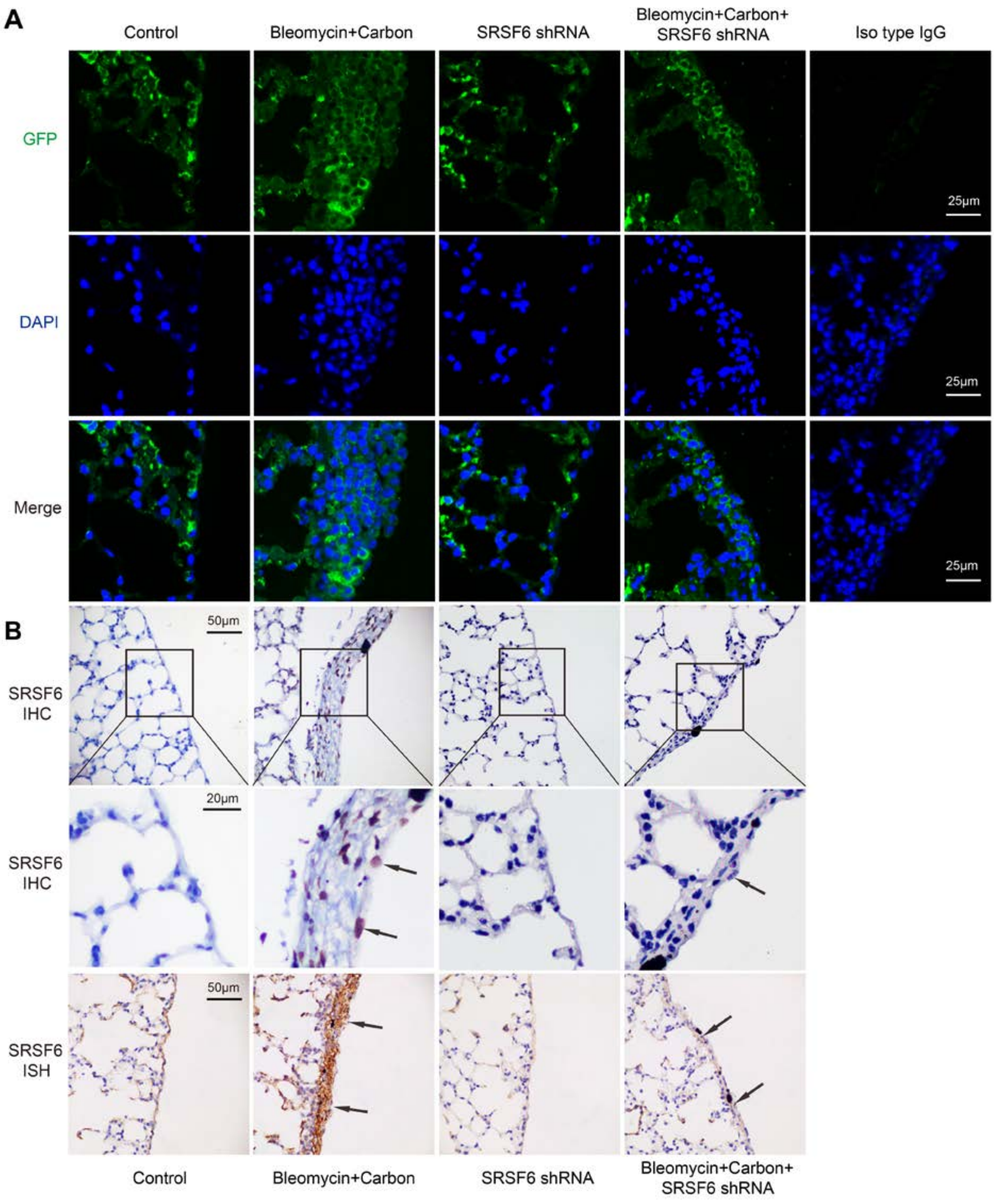

Supplemental Figure 7. SRSF6 shRNA transfection in mouse pleural fibrosis models. Pleural fibrosis in mice was induced by intra-pleural injections of bleomycin plus carbon particles. Lentivirus expressing GFP and shRNA directed against SRSF6 or scrambled sequence shRNA were administrated by intrapleural injection at a dose of 2×10^6 TU on days 4, 7 and 10. All mice were euthanized at day 21, and then tissues were taken for

immunohistochemical (IHC) staining and *in situ* hybridization histochemistry (ISH) staining to reveal SRSF6 protein and mRNA. (A) Representative images of GFP on visceral pleura from lung sections. Scale bar: 25 μm, Original magnification, ×200. (B) Representative images of SRSF6 IHC and ISH. Scale bar: 50 μm or 20 μm. Original magnification, ×400.

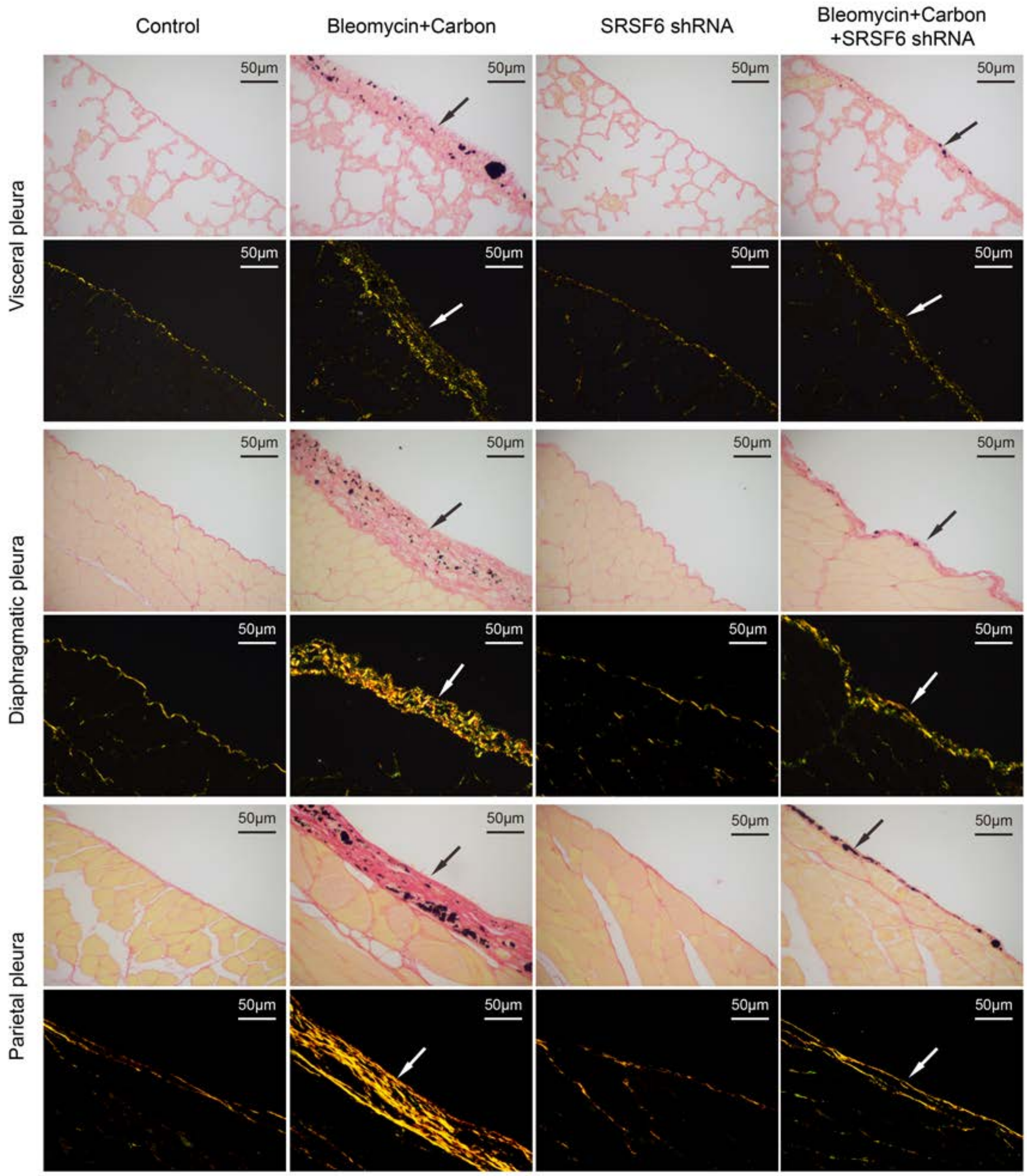

Supplemental Figure 8. SRSF6 knockdown by shRNA attenuated bleomycin induced pleural fibrosis in mouse model. Mouse pleural fibrosis model was induced by intra-pleural injections of bleomycin and carbon particles as the description in the Methods. Lentivirus expressing shRNA directed against SRSF6 or scrambled sequence shRNA were administrated by intrapleural injection at a dose of 2×10^6 TU on days 4, 7 and 10. All mice were euthanized at day 21. Visceral pleura from lung sections, parietal pleura from chest wall and diaphragm sections were stained with picrosirius red staining to reveal collagen. Images were taken under brightfield (upper) and polarized (lower) light. Scale bar: 50 μm. Original magnification, ×400.

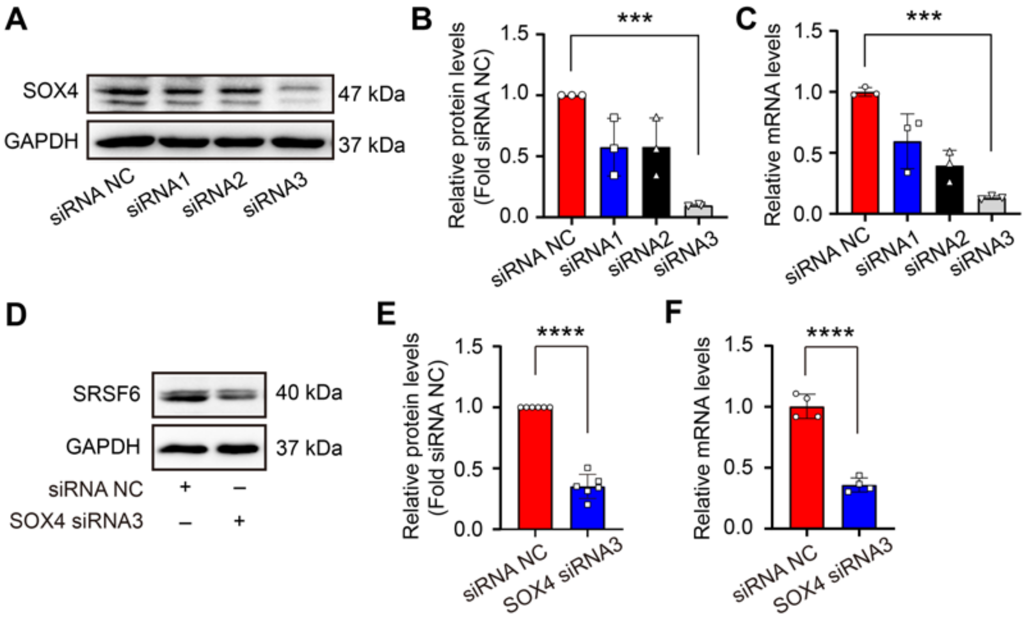

Supplemental Figure 9. SOX4 siRNA attenuated SRSF6 expression in PMCs. (A-C) Human PMCs were transfected with control or specific siRNA against SOX4 (siRNA1, siRNA2 and siRNA3) for 48 h. SOX4 protein and mRNA levels were assessed by western blotting (A, B), and RT-qPCR (C). Data are expressed as mean ± SEM of n individual experiments. n=3 (B), n=3 (C). ****P*< 0.001 versus control group (Student's t-test). (D- F) After transfection with SOX4 siRNA3 or negative control siRNA (siRNA NC), PMCs were incubated for 24 h, SRSF6 protein contents were measured by western blotting (D, E). mRNA levels were detected by RT-qPCR and normalized by the housekeeping gene GAPDH (F). Data are expressed as mean ± SEM of n individual experiments. n=6 (E), n=4 (F). *****P*<0.0001 versus control siRNA (Student's t-test).

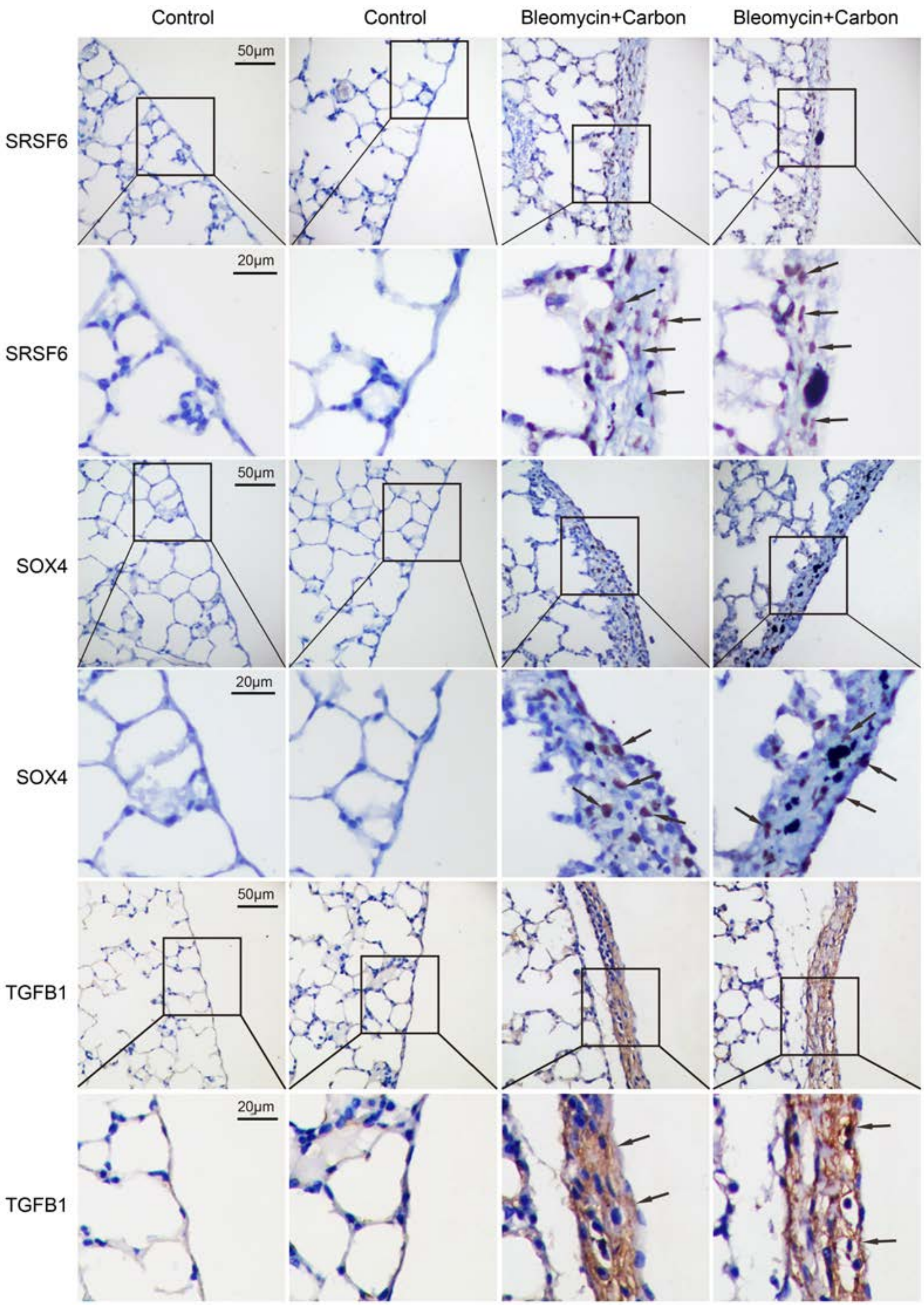

Supplemental Figure 10. SRSF6, TGFB1 and SOX4 protein changes in mouse pleural fibrosis model. Mouse pleural fibrosis was induced by intra-pleural injections of bleomycin plus carbon particles. Mice were euthanized at day 21, and then tissues were taken for immunohistochemical (IHC) staining of SRSF6, SOX4 and TGFB1 proteins. Scale bar: 20 μm or 50 μm. Original magnification, ×400.

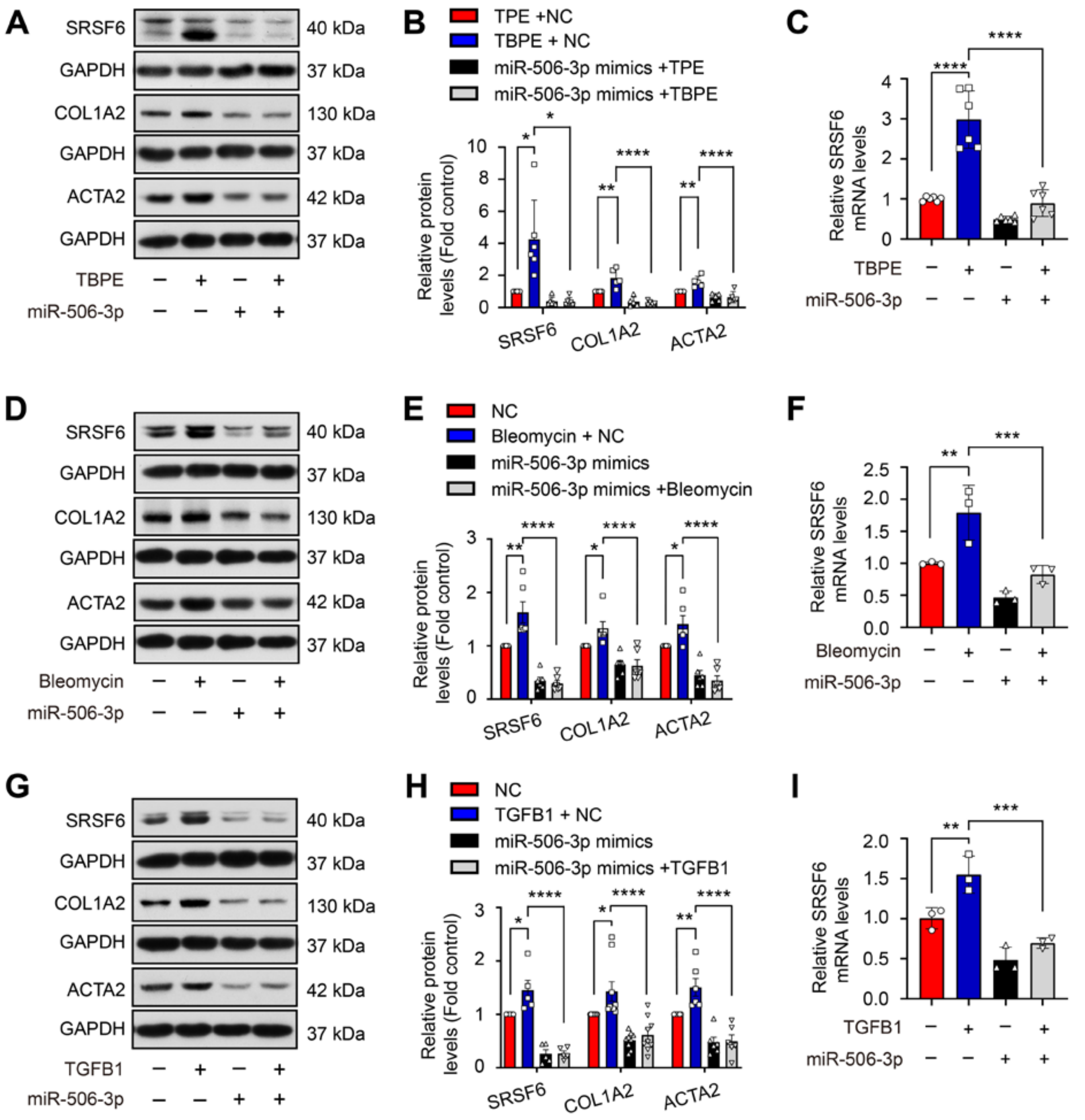

Supplemental Figure 11. Overexpression of miR-506-3p prevented TBPE-, bleomycin- and TGFB1-induced SRSF6, COL1A2 and ACTA2 synthesis in PMCs. After transfection with miR-506-3p mimics or negative control (NC), PMCs were incubated with TBPE or TPE (5%), bleomycin (0.2 μg/ml), or TGFB1 (5 ng/ml) for 48 h, after which intracellular SRSF6, COL1A2 and ACTA2 protein content was measured by western blotting (A, D, G). Changes in relative density of SRSF6, COL1A2 and ACTA2 to GAPDH were presented (B, E, H). mRNA expression of *SRSF6* was measured by RT-qPCR and normalized to GAPDH (C, F, I).

Data are mean ± SEM of n individual experiments. B: n=6 (SRSF6), n=5 (COL1A2, ACTA2); C: n=6; E: n=6; F, n=3; H: n=5 (SRSF6), n=9 (COL1A2), n=6 (ACTA2); I, n=3. *P<0.05, **P<0.01, ***P<0.001, ****P<0.0001 (One-way ANOVA followed by the Bonferroni's test).

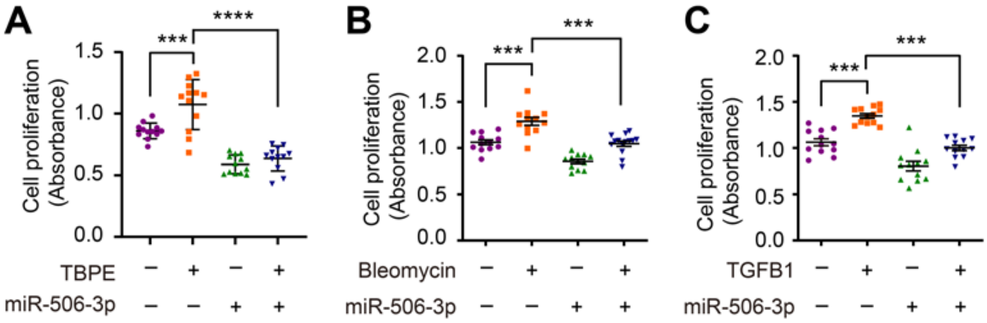

Supplemental Figure 12. Overexpression of miR-506-3p prevented TBPE-, bleomycin- and TGFB1-induced PMC proliferation. After transfection with miR-506-3p mimics or negative control, PMCs were incubated with TBPE (5%), bleomycin (0.2 μg/ml), or TGFB1 (5 ng/ml) for 24 h, after which cell proliferations was quantified using a CCK8 kit. Data are mean ± SEM of n individual experiments. n=12. ***P< 0.001, ****P<0.0001 (One-way ANOVA followed by the Bonferroni's test).

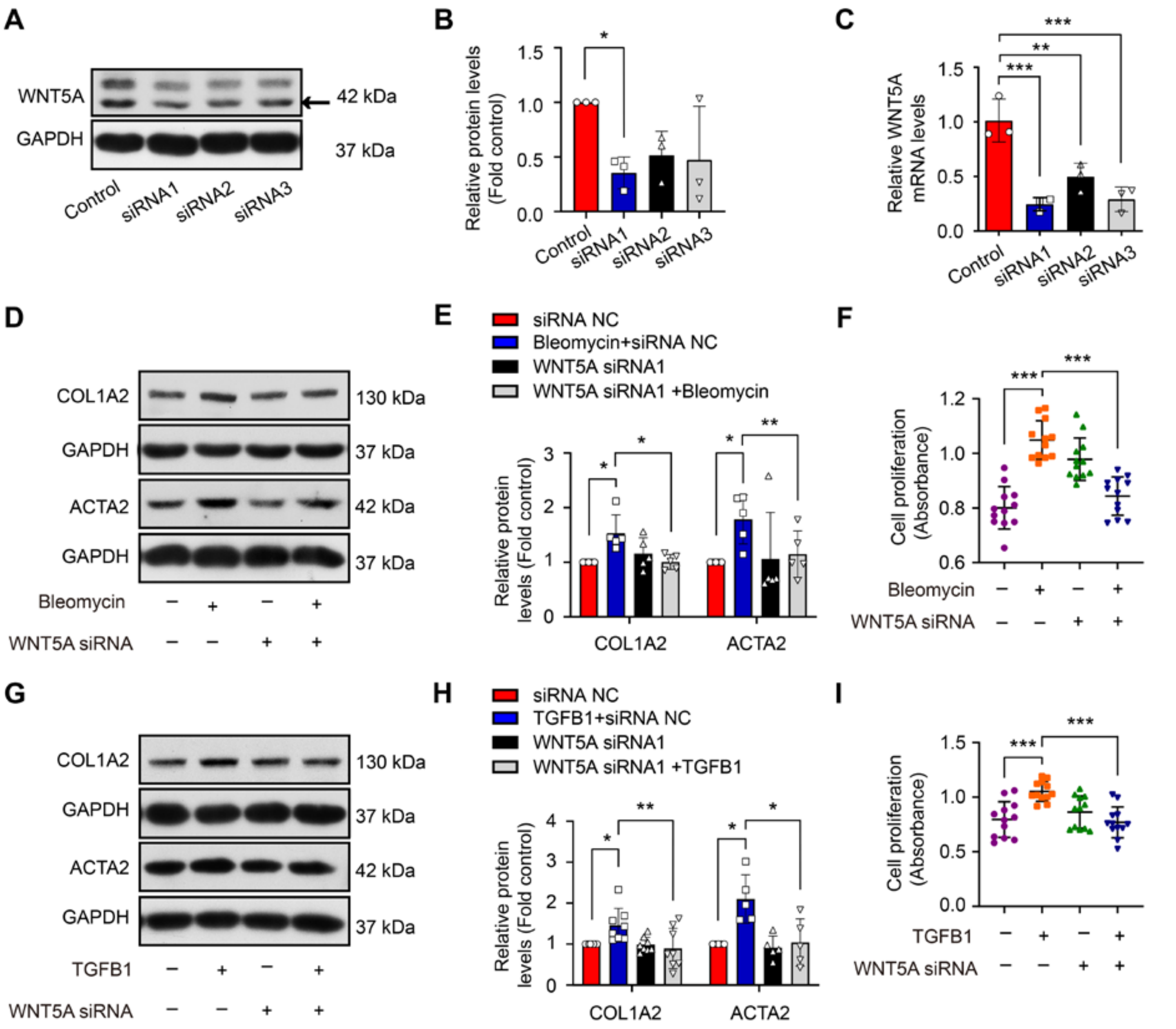

Supplemental Figure 13. Bleomycin- and TGFB1-induced COL1A2 synthesis and cell proliferation via activation of WNT5A in PMCs. (A-C) Human PMCs were transfected with control or specific siRNA against WNT5A (siRNA1, siRNA2 and siRNA3) for 48 h. WNT5A protein and mRNA knockdown levels were assessed by western blotting (A, B) and RT-qPCR (C). Data are mean ± SEM of n individual experiments, n = 3, **P*<0.05, ***P*<0.01, ****P*<0.001 versus control group (Student's t-test). (D-I) After transfection with WNT5A siRNA1 or negative control siRNA, PMCs were incubated with or without bleomycin (0.2 μg/ml) or TGFB1 (5 ng/ml) for 48 h, after which intracellular COL1A2 and ACTA2 protein contents were measured by western blotting (D, E, G, H). Cell proliferations were detected by

CCK8 kit (F, I). Data are expressed as mean ± SEM of n individual experiments, E: n=5; F: n=12; H: n=8 (COL1A2), n=5 (ACTA2); I: n=12. *$P<0.05$, **$P<0.01$, ***$P<0.001$ (One-way ANOVA followed by the Bonferroni's test).

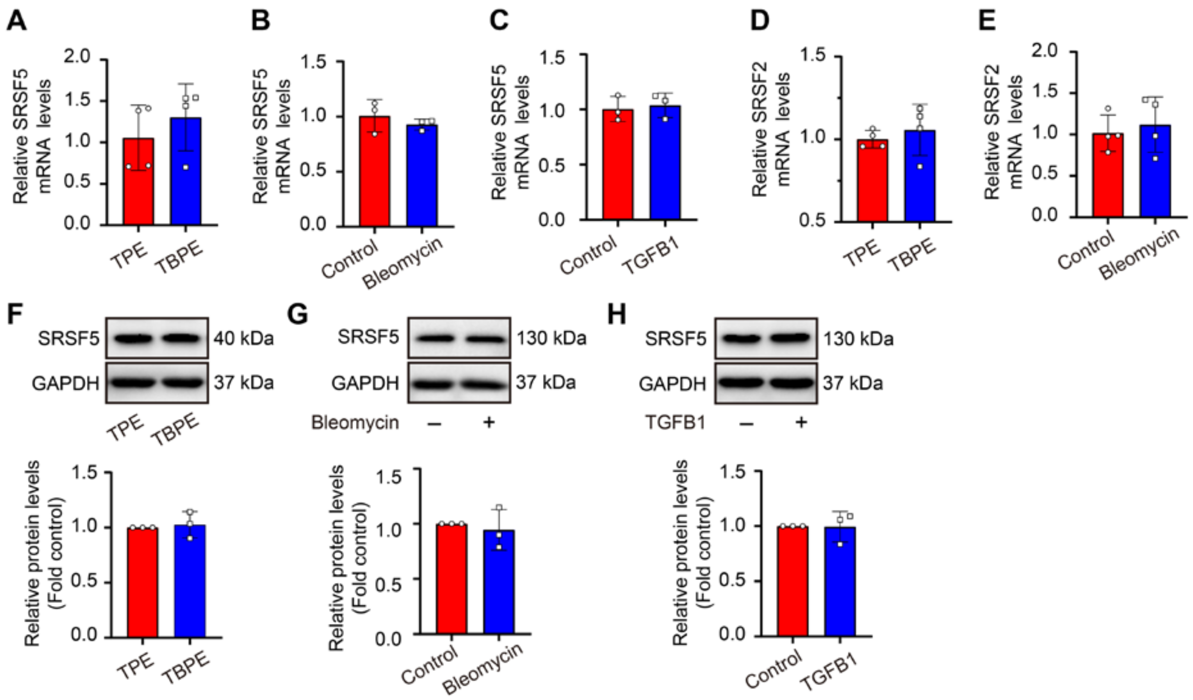

Supplemental Figure 14. Bleomycin did not change SRSF2 or SRSF5 in PMCs. Human PMCs were incubated with TBPE (5%), bleomycin (0.2 μg/ml) or TGFB1 (5 ng/ml) for 48 h, after which *SRSF5* and *SRSF2* mRNA and intracellular SRSF5 protein levels were measured by RT-qPCR and western blotting. (A-E) mRNA levels of *SRSF5* and *SRSF2* normalized to the housekeeping gene GAPDH. (F-H) Representative western blots, and changes in relative density of SRSF5 to GAPDH were presented. Data are expressed as mean ± SEM of n individual experiments. n=4 (A , D and E)，n=3 (B ,C, F, G and H). There is no difference between two groups in each panel tested by Student’s t-test.

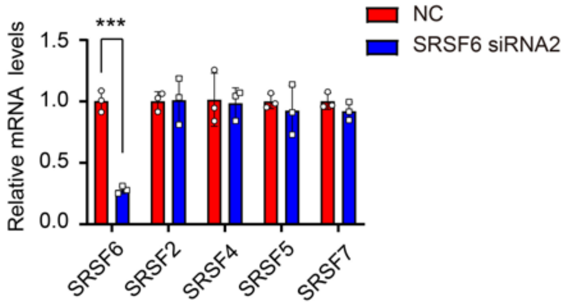

Supplemental Figure 15. SRSF6 siRNA did not block siRNA expression of SRSF2, SRSF4, SRSF5 or SRSF7 in PMCs. Human PMCs were transfected with specific siRNA against SRSF6 (SRSF6 siRNA2 in Figure 2) or negative control siRNA (NC siRNA) for 48 h. *SRSF6*, *SRSF2*, *SRSF4*, *SRSF5* and *SRSF7* mRNA levels were assessed by RT-qPCR. Data are expressed as mean ± SEM of n individual experiments. n=3. ****P*<0.001 versus negtive control siRNA (Unpair Student's t-test).

Supplemental Table 1. Clinical characteristics of patients with tuberculous pleural effusion and transudative pleural effusion

Characteristics	TBPE	TPE
N	12	9
Males/Females, n/n	10/2	5/4
Age, years		
Mean ± SD	35.5±17.0	68.6 ±13.7
Range	20-73	48-85

Data are presented as mean ± SD or range.

TBPE: tuberculous pleural effusion; TPE: transudative pleural effusion

Supplemental Table 2. Sequences of RT-PCR/qPCR primers, siRNA oligonucleotides and shRNA

Applications	Genes	Sequences (5′→3′)	Size (bp)
qPCR	(Human) SRSF6	F: TAACCTATGCGGATGCCCAC<br>R: CGACTCCTACTTCGTGACCG	220
qPCR	Mouse SRSF6	F: ACGAGCTCAACAGCAAGGAG<br>R: CCGACTGCTGTATCCACCTC	125
qPCR	Rat SRSF6	F: TAGGACGCCTGAGCTACAAC<br>R:TCCACGAAACCGTACCCATT	106
qPCR	COL1A2	F: CTGGTGAAGCTGGTCGTGAT<br>R: CCACGGTTTCCATGTTTGCC	175
qPCR	Rat COL1A2	F: CACTGTCCTTGTCGATGGCT<br>R: GGCAGGCGAGATGGCTTATT	90
qPCR	ACTA2	F: GAGCGTGGCTATTCCTTCGT<br>R: GCCCATCAGGCAACTCGTAA	154
qPCR	Rat ACTA2	F: AGCATCCGACCTTGCTAACG<br>R: AGAGTCCAGCACAATACCAGTTG	167
qPCR	SOX4	F: ACCAACAATGCCGAGAACACG<br>R: CTCGATCTGCGACCACACCAT	207
qPCR	GAPDH	F: TGGCTACAGCAACAGGGTGG<br>R: GGTACATGACAAGGTGCGGCT	229
qPCR	Mouse GAPDH	F: AGGTCGGTGTGAACGGATTTG<br>R: GGGGTCGTTGATGGCAACA	95
qPCR	Rat GAPDH	F: TGCCACTCAGAAGACTGTGG<br>R: TTCAGCTCTGGGATGACCTT	129
qPCR	SRSF2	F: GCACGAAGGTCCAAGTCCAA<br>R: TCCGAGCAGCACTCCTAAT	293
qPCR	SRSF4	F:AGCTGGCAAGACCTAAAGGATT	261

		R: ACGGGAATGTCTGCTTCGAG	
qPCR	SRSF5	F: GGTCAAGAAGCAGGTCTCGAT<br>R: GATTTGCGACTACGGGAACG	79
qPCR	SRSF7	F: ACGAAGGTCAAGGTCAGCATC<br>R: CGACTGCTTCTTGGTCGTGA	174
GE	AHR	F: GCAGCGCCAACATCACCTAC<br>R: AGCCAAACGGTCCAACTCTGT	143
GE	NREP	F: GAGGCTTCCTAAGGGAAGACTT<br>R: AAGTGGAGGTAACTGATTCTTGG	132
GE	WNT5A	F: ACATCGGAGAAGGCGCGAAG<br>R: CGCTCACCGCGTATGTGAAG	153
GE	SMAD5	F: CTAGAAGATAATGGTTAGGCAAGTG<br>R: TGTGGGCAGGTAGATACAAACA	135
GE	RNF146	F: AAGCGGTGTGCTCTTTGTCG<br>R: CGCTCATCGTACTGCCACCA	155
GE	SENP1	F: CGGGTGGAGTTTGGACTGAT<br>R: AATGCGGTACAACGGGAGAG	230
GE	MAPK14	F: CAGGAGAGGCCCACGTTCTA<br>R: AGCACACACAGAGCCATAGG	114
ChIP	Site 1	F: TCACTCCTCCCAGTCTCC<br>R: GTGGCAATCCTACGCTCA	305
ChIP	Site 2	F: CAATCAGCAGGATGTAGGC<br>R: GACTCCAACCTGCCTGAA	129
siRNA	Human SRSF6 (siRNA1)	GCGUCUACAUAGGACGCCUGAGCUA<br>UAGCUCAGGCGUCCUAUGUAGACGC	/
siRNA	Human SRSF6 (siRNA2)	CCUGUUCGUACAGAAUACAGGCUUA<br>UAAGCCUGUAUUCUGUACGAACAGG	/
siRNA	Human Control	UUCUCCGAACGUGUCACGUTT	/

	(NC siRNA)	ACGUGACACGUUCGGAGAATT	
shRNA	Mouse SRSF6 (shRNA1)	ccAGATCAAGTTCCAGAGATT	/
shRNA	Mouse SRSF6 (shRNA2)	cgAACAAATGAGGGTGTGATT	/
shRNA	Mouse SRSF6 (shRNA3)	cgTACAGAGTACAGGCTTATT	/
shRNA	Mouse shRNA Negative Control	TTCTCCGAACGTGTCACGT	/
siRNA	Rat SRSF6 (siRNA1)	GGAUACAGCAGUCGGAGAATT<br>UUCUCCGACUGCUGUAUCCTT	/
siRNA	Rat SRSF6 (siRNA2)	GCAGGAAUAUACGGCUUAUTT<br>AUAAGCCGUAUAUUCCUGCTT	/
siRNA	Rat SRSF6 (siRNA3)	GCUUCAAGGUCCCGUUCUATT<br>UAGAACGGGACCUUGAAGCTT	/
siRNA	Rat Control (NC siRNA)	UUCUCCGAACGUGUCACGUTT<br>ACGUGACACGUUCGGAGAATT	/
siRNA	Human WNT5A (siRNA1)	GAAGUCCAUUGGAAUAUUATT<br>UAAUAUUCCAAUGGACUUCTT	/
siRNA	Human WNT5A (siRNA2)	CUAGUGGCUUUGGCCAUAUTT<br>AUAUGGCCAAAGCCACUAGTT	/
siRNA	Human WNT5A (siRNA3)	GCCAGUAUCAAUUCCGACATT<br>UGUCGGAAUUGAUACUGGCTT	/
siRNA	Human SOX4 (siRNA1)	GCAAACCAACAAUGCCGAGTT<br>CUCGGCAUUGUUGGUUUGCTT	/
siRNA	Human SOX4 (siRNA2)	GCGACAAGAUCCCUUUCAUTT<br>AUGAAAGGGAUCUUGUCGCTT	/
siRNA	Human SOX4	ACCACCACUCGCUGUACAATT	/

(siRNA3)	UUGUACAGCGAGUGGUGGUTT

Application: quantitative RT-PCR (qPCR), Validation of gene expression (GE), Chromatin immunoprecipitation (ChIP) and siRNA oligo sequences (siRNA).

Supplemental Table 3. miRNA sequences for hsa-miR-506-3p

miRNA name	miRNA Sequences (5′→3′)
Hsa-miR-506-3p mimics	Sense: UAAGGCACCCUUCUGAGUAGA<br>Anti-sense: UCUACUCAGAAGGGUGCCUUA
Mimics negative control (mimics NC)	Sense: UCACAACCUCCUAGAAAGAGUAGA<br>Anti-sense: UACUCUUUCUAGGAGGUUGUGAUU
Hsa-miR-506-3p inhibitors	UCUACUCAGAAGGGUGCCUUA
Inhibitors negative control (Inhibitors NC)	UCUACUCUUUCUAGGAGGUUGUGA
Hsa-miR-506-3p RT primer	GTCGTATCCAGTGCAGGGTCCGAGGTATTCGCAC-TGG ATACGACTCTACT
Hsa-miR-506-3p primer	F: CGCGTAAGGCACCCTTCTG<br>R: AGTGCAGGGTCCGAGGTATT
